# Effects of Orthographic Consistency on Bilingual Reading: Human and Computer Simulation Data

**DOI:** 10.3390/brainsci11070878

**Published:** 2021-06-30

**Authors:** Eraldo Paulesu, Rolando Bonandrini, Laura Zapparoli, Cristina Rupani, Cristina Mapelli, Fulvia Tassini, Pietro Schenone, Gabriella Bottini, Conrad Perry, Marco Zorzi

**Affiliations:** 1 Psychology Department, University of Milano-Bicocca, 20126 Milano, Italy; eraldo.paulesu@unimib.it (E.P.); laura.zapparoli@unimib.it (L.Z.); cristina.rupani@gmail.com (C.R.); 2 IRCCS Istituto Ortopedico Galeazzi, 20161 Milano, Italy; 3 School of Medicine and Surgery, University of Milano-Bicocca, 20126 Milano, Italy; cristina.mapelli1@gmail.com; 4 Civica Scuola Interpreti e Traduttori, 20144 Milano, Italy; f.tassini@fondazionemilano.eu (F.T.); pietro.schenone@gmail.com (P.S.); 5 Department of Brain and Behavioural Sciences, University of Pavia, 27100 Pavia, Italy; gabriella.bottini@unipv.it; 6 Cognitive Neuropsychology Centre, ASST “Grande Ospedale Metropolitano” Niguarda, 20162 Milano, Italy; 7 Department of Psychology, The University of Adelaide, Adelaide 5005, Australia; conrad.perry@adelaide.edu.au; 8 Department of General Psychology and Padova Neuroscience Centre, University of Padova, 35131 Padova, Italy; 9 IRCCS Ospedale San Camillo, 30126 Venice-Lido, Italy

**Keywords:** reading, bilingualism, orthography, orthographic regularity, language, CDP++

## Abstract

English serves as today’s lingua franca, a role not eased by the inconsistency of its orthography. Indeed, monolingual readers of more consistent orthographies such as Italian or German learn to read more quickly than monolingual English readers. Here, we assessed whether long-lasting bilingualism would mitigate orthography-specific differences in reading speed and whether the order in which orthographies with a different regularity are learned matters. We studied high-proficiency Italian-English and English-Italian bilinguals, with at least 20 years of intensive daily exposure to the second language and its orthography and we simulated sequential learning of the two orthographies with the CDP++ connectionist model of reading. We found that group differences in reading speed were comparatively bigger with Italian stimuli than with English stimuli. Furthermore, only Italian bilinguals took advantage of a blocked presentation of Italian stimuli compared to when stimuli from both languages were presented in mixed order, suggesting a greater ability to keep language-specific orthographic representations segregated. These findings demonstrate orthographic constraints on bilingual reading, whereby the level of consistency of the first learned orthography affects later learning and performance on a second orthography. The computer simulations were consistent with these conclusions.

## 1. Introduction

When learning a second language, we not only rely on oral communication, but also on reading. The simpler the relationship between orthography and sound, the more efficient reading should be in supporting the learning process. As it happens, English, the most common second language [1] due to its relevance in cross-cultural communication and business, has a relatively inconsistent orthography that makes the acquisition of reading skills harder even in native English children [2]. It also makes the symptoms of dyslexia more severe than those seen in German [3] and Italian [4] dyslexics, despite the very similar brain abnormalities documented in dyslexics from different cultures [4,5]. A comparatively lower reading speed can still be found in adult English-proficient readers when compared with similar readers of consistent orthographies, with larger neural labor in the brain areas devoted to the integration of visual input with word sounds [6]. More generally, the psycholinguistic literature has approached the degree of regularity of the correspondence between print and sound in terms of “orthographic depth”. In this regard, a “shallow” orthography is characterized by a direct and unequivocal mapping between graphemes and phonemes, while in a “deep” orthography, a given letter could represent different phonemes, and different letters may be used to represent a given phoneme [7]. Of course, orthographic depth does not imply a dichotomy (shallow vs. deep), but instead, a continuum in which orthographies can be located based on their orthographic regularity. Accordingly, differences in reading times attributable to orthographic regularity have been explored in the comparison between Hebrew, English, and Serbo-Croatian (in order of transparency) [8], between English and French (French being more regular than English) [9] and between Finnish, Hungarian, Dutch, Portuguese, and French (in order of depth) [10], with reading in more irregular orthographies being typically slower than in more regular orthographies. If one considers the challenges that orthographic depth imposes to print-to-sound mapping during reading, bilingualism in the case of languages with different orthographic transparency constitutes an interesting scenario. In this regard, it has been suggested that some degree of first-to-second-language transfer of orthographic decoding mechanisms can take place [11,12,13], at least when the involved languages share the same alphabetic system (see [14] for a review). More specifically, recent evidence suggests that orthographic regularity of the first orthography affects the way in which reading in the second orthography is carried out [13,15].

However, the extent to which the effects of orthographic regularity on reading interact with the order in which languages are learnt is far from clear. The present study aims to address this topic in the context of Italian-English and English-Italian bilingualism (Italian orthography being more regular than the English one), while exploring how easily orthographic decoding knowledge coming from L1 can be smoothly integrated with the specific demands of L2. Previous seminal work on phonological auditory segmentation tasks in English and French bilinguals showed that French-dominant bilinguals have syllable-based segmentation of auditory language; on the other hand, English-dominant bilinguals show a stress-based segmentation. This has led to the provocative conclusion that bilingual speakers master one and only one auditory segmentation strategy [16,17] (see also [18] for an alternative account). Would similar conclusions also apply for reading aloud? On the other hand, would long-lasting bilingualism modify the patterns or cross-cultural differences in reading performance seen when comparing mono-lingual readers [6,8]? Even more crucially: what is the role of orthographic regularity in these phenomena?

### 1.1. Current Views on Monolingual and Bilingual Reading with Orthographies of Different Consistency

We previously proposed that native readers of the English orthography may use a broader set of weak associations between spellings and sounds than native readers of other alphabetical orthographies [6]. Indeed, for English, one can count up to 1120 ways of representing the 40 sounds (phonemes) of the language by means of different letters or letter combinations (graphemes) [1]; the mappings between graphemes, phonemes, and whole word sounds are often ambiguous and inconsistent to such an extent that even cultivated English speakers may find it hard to tell which word of minimally distinctive pairs, such as cough/bough or clove/love, are those that obey a general rule of spelling. By contrast, Italian has a much smaller set of graphemes to represent its 25 phonemes and the mappings from graphemes to phonemes are consistent.

The difference between English and Italian orthographies is also attested by computational models of reading that acquire the knowledge of spelling–sound correspondences through associative learning on large-scale word corpora (see [19] for English, and [20] for Italian). Simulations have shown that learning occurs faster and with less error in the Italian compared to English model, and the spelling–sound mapping in the Italian model is more compact in terms of connection weights (see Supplementary Materials, Figure S1).

Of course, not all grapheme to phoneme mappings are equally (in) frequent in English. It thus may be the case that word or pseudo-word reading with stimuli made of high frequency spelling–sound relationships show less of a difference across languages. However, using stimuli formed of high frequency bigrams does not cancel out cross-language differences in the decoding speed of monolinguals [6] (see also [8]).

Would this difference remain for highly proficient bilinguals while reading each set of stimuli with the language-specific spelling–sound correspondences? Remarkably, in monolinguals, it is virtually impossible to tell whether cross-linguistic differences in reading speed are simply due to the structural regularity of the orthography being read, or rather if they are due to the readers’ reading system being “tuned” to decode that orthography. Of course, one could administer monolinguals with reading tasks with stimuli from different languages, but any theoretical inference would be limited by cross-linguistic differences in reading proficiency.

Conversely, in highly proficient late-acquisition bilinguals, the effects of language and “orthographic tuning” (operationalized in terms of transparency of the first learnt orthography) can be disentangled, while maintaining an equally optimal level of performance at a behavioral level. Of course, this does not rule out the possibility that the amount of familiarity with certain words could impact on the estimation of the effects of language and of first orthographic experience. Highly proficient bilinguals also provide the unique opportunity to explore reading times for novel words (pseudo-words) while using with the orthographic specific decoding rules, at variance with what can be achieved with monolinguals.

In this regard, it would be harder to explain cross-cultural differences in reading speed for pseudo-words in bilinguals of comparably high proficiency in both languages. If differences still existed, with Italian bilinguals reading pseudo-words faster than English bilinguals, this may suggest that Italian bilinguals simply have a better command of sub-lexical reading strategies [21]. Alternatively, the learning of the inconsistent orthography might have the reverse effect, slowing down Italian bilinguals while reading Italian words and pseudo-words. In this case, one might speculate that learning English causes an increase in the set of spelling–sound mappings: if shared across both languages, such set of mappings would make reading Italian less efficient.

### 1.2. Bilingual Reading: One or Multiple Orthographic Systems?

Contemporary models of bilingual word recognition have settled on the assumption that the bilingual language system is organized in an integrated lexicon that is accessed in a language-non-selective manner [22,23,24,25]. However, this does not necessarily imply that all components of the reading network are shared across the first and second language. Moreover, the development of orthographic lexical representations depends—at least in the initial stages of reading—on the ability to associate letters and groups of letters with sounds [26,27]. Hence, the nature of grapheme–phoneme correspondences, which are typical of the language(s) to which the individual is exposed, determines how novel words (or pseudo-words) are read and contributes to shaping and organizing orthographic representations at the lexical level as well. Accordingly, it was shown that the sensitivity to language-specific orthographic sub-lexical features plays an important role in guiding the access to language-specific orthographic and lexical representations in bilinguals [28]. In more general terms, Lallier and Carreiras [29] suggested that bilingual reading development varies according to the specific combination of orthographies learned. Assuming that more transparent orthographies are associated with more efficient, even if not exclusive [30], processing of small chunks of information such as graphemes (i.e., a small grain size; see [21]), while more opaque orthographies require the inevitable simultaneous processing of bigger chunks of information (i.e., a larger grain size), the concurrent exposure to different orthographies during reading development may induce a “grain size accommodation”. In other words, the type of reading strategy preferred by bilinguals would be a hybrid between those that would be used by monolingual readers of the different orthographies. Nevertheless, this hypothesis is focused on early acquisition bilinguals learning two orthographies simultaneously. No predictions are made on late acquisition bilingualism and on how learning a specific set of spelling–sound mappings impacts the subsequent learning of an orthography with a different degree of transparency and the ensuing effects on the internal organization of the bilingual reading system.

### 1.3. Aims of the Study

Our study was designed to test whether long-term bilingualism modulates the behavioral differences for reading that are documented in monolinguals of different cultures [6,19,31,32]. We assessed whether the order whereby a consistent and an inconsistent orthography are learned matters.

Previous observations in monolinguals suggested that learning a relatively irregular orthography, such as the English one, leads to the use of a broader set of weak associations between spellings and sounds than the one required by more regular orthographies such as the Italian one [6]. On the other hand, it has been suggested that the way with which graphemes and phonemes are associated during reading acquisition shapes the development of the reading system [26,27].

In this regard, we tested the possibility that the effect of first orthographic exposure could have a different impact on reading stimuli from two languages characterized by a different degree of transparency. The available literature provides little (if any) predictions in this direction, mainly due to the lack of studies in which both Language A-to-B and B-to-A bilingualism were simultaneously explored in the context of orthographies with a different degree of regularity. Nevertheless, previous literature suggests that plasticity diminishes overtime [33]. Accordingly, learning a second orthography should trigger less dramatic changes to the cognitive system than learning the first orthography. On the other hand, previous literature suggests that—from a computational point of view—a learning process whose training set is initially small (and increases in size as learning proceeds) is more efficient than a learning process that deals with a large training set from the beginning [34]. In our case, Italian orthography—due to its greater regularity—implies a smaller set of grapheme–phoneme rules to be learnt that English. Therefore, we expect that learning a relatively irregular orthography such as English after a relatively regular one such as Italian allows the graceful integration of strong and efficient grapheme–phoneme associations typical of a more regular orthography with the weak ones required by the more irregular orthography. Conversely, we anticipate that first “tuning” the reading system to a relatively irregular orthography may not allow to efficiently integrate weak and strong grapheme–phoneme mappings, thus limiting the possibility to catch up fully with the simplicity of a second, more consistent orthography. These effects should emerge as a greater difference in reading times between L1-Italian and L1-English bilinguals while reading Italian stimuli than while reading English stimuli.

To address these issues, we studied well-balanced Italian-English and English-Italian bilinguals (see Table 1) with long-lasting bilingual experience, whereby the second language and orthography were practiced on a daily basis for at least 20 years.

We assessed reading speed and accuracy in naming single words and pseudo-words in English and Italian, using language-specific high-frequency regular orthographic stimuli. In this regard, performance on pseudo-words was expected to be particularly informative because it is unconfounded from any lexical factor, including individual familiarity with the orthographic stimuli. To further test the face validity of our behavioral observations, we complemented our investigation using computer simulations of sequential learning of the English and Italian orthographies based on the connectionist dual process (CDP++) model of reading aloud [27], a large-scale computational model available for both English [19] and Italian languages [20].

## 2. Materials and Methods

The study was approved by the local ethics committee (Comitato Etico Milano Area 3. Protocol ID: 3216) and was conducted following the principles of the Declaration of Helsinki. All participants gave their written, informed consent to take part in the study.

### 2.1. Participants

Eighteen Italian-English (L1-Italian) bilinguals (M = 9, F = 9; mean age = 34.611; SD = 14.561; mean age of second language acquisition: 7.250 years; SD = 5.056) and 22 English-Italian (L1-English) (M = 12, F = 10; mean age = 42.364, SD = 11.701; age of second language acquisition: 12.000 years; SD = 9.730) bilinguals with intensive daily exposure to both languages since at least 20 years ago were tested with reading tasks, during which accuracy and voice reaction times were recorded, and with a number of control tasks (see Table 1 for demographic data and information on bilingual experience). All participants were living in Italy at the time of testing.

These bilinguals can be labelled as high-proficiency late acquisition bilinguals, not to be confused with subjects who are brought up in a bilingual environment from birth and who nevertheless may be seldom exposed to two orthographies from the beginning of their schooling or are not necessarily practicing more than one orthography in their daily life.

There were seven professional interpreters/translators in the L1-English group and five in the L1-Italian group. The other participants were either language teachers, ex-pupils of the Milan Council School for Interpreters and Translators, university academics not involved in language teaching, or professionals in other fields, all fulfilling the criteria of daily exposure to both languages for at least 20 years. The two groups were balanced on linguistically important factors including intensity of daily exposure to both oral languages, age at which they became bilinguals, duration of their bilingualism and proficiency in Italian-to-English and English-to-Italian translation skills (see Supplementary Materials for further information: Tables S4 and S6). On the other hand, the daily reading experience, as self-assessed by the participants in a questionnaire, reflected a simple L1 preference (see Table 1). Further demographic variables (e.g., age; M/F ratio) were also not significantly different across groups.

### 2.2. Experimental Design

The study consisted of experimental behavioral tasks in which the reading speed and reading accuracy were measured. The protocol also included control tasks that explored behaviors that are relevant for reading speed measurements and task switching (see also the Supplementary Materials on the Stroop test). The order of the tasks was counter-balanced and pseudo-randomized across subjects to avoid language-specific order effects in any task involving an Italian and an English parallel form (e.g., controlled word retrieval; word reading).

### 2.3. Control Behavioral Tasks

- Simple Vocal Reaction Times for Visual Stimuli: Participants were asked to say “PRONTI” as quickly as possible when a black cue circle appeared in the center of a computer screen. There were two different blocks with 25 stimuli each. The inter-stimulus interval was randomized around a mean value of 1.5 s (SD: 0.5 s; range: 600–2400 ms). All the participants performed this task twice: before and after the reading tasks.
- Articulation Speed: Participants were asked to pronounce, for fifteen seconds, the pair of translingual words “TENNIS-POLO” as quickly as possible.
- Verbal Semantic Fluency: Participants were asked to generate, for one minute, in English and in Italian, as many words as possible belonging to a semantic category (animals and fruit). For each subject, and separately for either language, the number of produced words in the two categories (animals and fruit) was averaged.

### 2.4. Experimental Behavioral Tasks

Subjects were asked to read aloud, as quickly as possible, single words and pseudo-words presented in Italian and in English (See Table 2 for further information). All the tasks were administered using the SuperLab Pro program (Experimental Lab Software, version 2.0.2. Copyright Cedrus Corporation, 1996–2003). All the vocal reaction times were collected using a portable PC and a connected microphone. The voice key triggers were validated by recording the latencies to a continuous tone in response to each visual stimulus: this, by definition, gave 1 msec latency—the minimum recordable differences by the device—to each of the presented stimuli. This calibration was used before each session. The word reading tasks were performed either in a blocked-design or in a mixed presentation format, containing Italian and English words in the same block, in separate runs. Accuracy was determined online by the examiners (while readers were reading).

The stimulus set was derived from the same pool of stimuli adopted by [6], in order to maintain the highest possible level of comparability with previous data on monolingual reading.

- English word reading: subjects were asked to read two blocks of 20 English regularly spelled words aloud. All the stimuli, selected from the 7500 English most frequent words, were disyllabic and stressed on the first syllable.
- Italian word reading: subjects were asked to read two blocks of 20 Italian words each aloud. All the stimuli, selected from the 7500 most frequent Italian words, were disyllabic and stressed on the first syllable.
- English pseudo-word reading: subjects were asked to read two blocks of 20 English pseudo-words aloud. All the stimuli were created by modifying one or two phonemes of English disyllabic words, without changing the syllabic structure of the original word (e.g., “paper = paber”). Subjects were invited to read these stimuli as if they were rare words from English.
- Italian pseudo-word reading: subjects were asked to read two blocks of 20 Italian pseudo-words each aloud. All the stimuli were created by modifying one or two phoneme(s) of Italian disyllabic words, without a change of the syllabic structure of the original word (e.g., “testa = tesca”). Subjects were invited to read these stimuli as if they were rare words from Italian.
- Mixed language reading tasks: A fresh set of 60 regular disyllabic words (30 Italian and 30 English words) were also presented in a randomized order. The reading task was performed in two separate runs of 30 stimuli each.

All stimuli and their psycholinguistic characteristics (bigram frequency; neighborhood size; word frequency) are reported in the Supplementary Materials, Table S5. As far as the accuracy of pseudowords is concerned, all the possible acceptable pronunciations of each pseudoword were determined by two professional interpreters (P.S. and F.T.). The pronunciation of a pseudoword was considered inaccurate if a subject’s utterance was not among the possible pronunciations of that stimulus.

### 2.5. Data Analysis

The analyses of the experimental tasks reported here are focused on reaction times, as the accuracy was at ceiling in all cases (see Supplementary Materials, Table S8) with only occasional reading errors (>90% accurate trials in all subjects). Of the overall 8800 data points of experimental tasks, 347 (3.9%) were discarded due to incorrect detection of voice onset times. A total of 68 trials (0.8%) were further discarded due to inaccurate readings, thus leaving a total of 8385 data points. For each analysis, in order to exclude that extreme values could bias our analyses, reaction times located three or more standard deviations from the overall mean were excluded. This led to the exclusion of further 11 trials (0.3%) in the analysis for word stimuli presented in blocked order, five trials (0.1%) in the analysis for pseudo-word stimuli presented in blocked order, and 13 trials (0.2%) in the analysis for word stimuli presented in blocked and mixed order.

Data were analyzed by means of linear mixed-effects models in which the RTs (log-transformed, in order to obtain a better approximation to a normal distribution than raw RTs; see Supplementary Materials, Figure S2) were the dependent variable [38]. Subjects and stimuli were modelled as random intercepts. This random structure was preferred over a maximal one [39] because with the latter, none of the adopted models could reach algorithm convergence. These analyses were conducted by means or the R software (version 4.0.3) and the lme4 package [40]. Where necessary, interaction effects were further explored by means of a planned comparison—run by means of the Phia package [41]—testing the hypothesis that the difference in reading times between L1-Italian and L1-English bilinguals is bigger with Italian stimuli than with English stimuli.

Data of control tasks were analyzed with *t*-tests (or equivalent non-parametric tests depending on the data distribution) and ANOVAs followed by Bonferroni-corrected pairwise comparisons. These analyses were performed by means of the software Jamovi (version 0.9.2.9).

### 2.6. Control over Possible Confounding Effects Related to Stimuli

Initial phoneme, frequency, bigram frequency and orthographic neighborhood size were included as covariates in control models, to exclude that the interactions of interest (namely, the language-by-group interaction in the blocked tasks and the language-by-group-by-task interaction in the blocked-mixed reading task) could be cancelled out after controlling for more general orthographic and phonemic phenomena. In no case did the inclusion of these variables have an impact on the significance of the crucial interaction effects. These analyses are reported in the Supplementary Materials (Supplementary Methods and Results, Tables S1–S3).

### 2.7. Computer Simulations

Learning to read in the L2 orthography was simulated using the most recent developmental version of the CDP++ model of reading [42]. After training the L1 models (one for each language), L2 models were created by taking the connection weights from the spelling–sound mapping network of the L1 models and placing them in the corresponding network of the L2 models for all graphemes that were shared across the two languages. For any shared grapheme, weights were taken for the connections to all phonemes that were relatively close in terms of features to the L1 phoneme and copied in the L2 network (see Supplementary Materials for details: Supplementary Methods and Results, Figures S4 and S5, and Section). This procedure seeded the L2 networks with information from the L1 network. Learning was then started for the L2 models in the same way as for the L1 models, except for a smaller learning rate. L1 and L2 models were tested at various points during learning using the same stimuli (English or Italian) read aloud by the human participants. As standard practice in computational modelling of reading aloud [19], model performance (accuracy and reading times) was assessed at the level of individual items and submitted to statistical analyses. See the Supplementary Materials for further details on models and simulations.

It should be pointed out that the L2 networks are networks that were trained on the second orthography after being seeded from the first orthography (here and henceforth: by first and second orthography (O1 and O2), we specifically refer to the written form of the first and second acquired language, respectively.): after training, the L2 networks “read” the stimuli used for our human data only for the second orthography measurements. Hence, the L2 networks capture the learning transition from one orthography to the second. Accordingly, we use the wording “equivalent” between human bilinguals and L2 two networks in as much as the L2 networks learned a second orthography. As the computational model lacks an orthography specific recognition module that bilinguals are hypothesized to have, and cannot switch from one orthography to the other, the L2 networks were tested only on the blocked presentation of the L2 orthographic stimuli.

## 3. Results

The empirical tests of the questions spelled out in the introduction led to the following observations (see Table 1 and Table 3 and the methods section).

### 3.1. Control Tasks

First, the two groups were well matched for their skills in the elementary control tasks such as the articulation speed and vocal reaction times to a simple visual stimulus.

Second, ease of word retrieval and possible L1-related differences in the two groups were assessed with a semantic fluency task, and analyzed with a 2 (group: L1-Italian vs. L1-English bilinguals) by 2 (language: Italian vs. English) ANOVA with the group as a between-subjects factor and language as a within-subjects factor.

**Figure 1:**
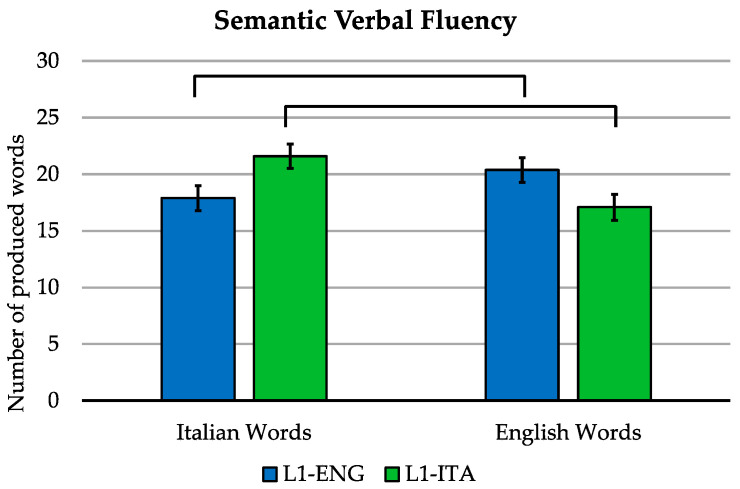
Semantic verbal fluency. Mean number of English and Italian words produced during the semantic verbal fluency tasks by L1-Italian and L1-English bilinguals. Error bars indicate the standard error of the mean.

Figure 1 illustrates these results. Both the main effects of the language [F(1,38) = 3.480, *p* = 0.070] and group [F(1,38) = 0.020, *p* = 0.888] were not significant. However, there was a significant language-by-group interaction [F(1, 38) = 41.413, *p* < 0.001]. Pairwise comparisons revealed that Italian bilinguals produced a greater number of Italian words than English words [t(38) = 5.596, *p* < 0.001], while for the English bilinguals, the opposite pattern was true [t(38) = −3.406, *p* = 0.009].

To summarize, as expected, the controlled word retrieval task revealed a persisting advantage for word production in L1 for each group of bilinguals, in line with what is observed in highly proficient bilinguals [43].

Third, with the specific reading-aloud tasks, we found that a simple language effect, as for controlled word retrieval, could not explain the reading behavior observed; this is best explained by an interaction between the consistency of the orthography and order of acquisition. The results presented below are based on the log-transformed reading reaction times of correct trials. Accuracies were >0.90 for all subjects.

### 3.2. Word Reading (in Blocks)

**Figure 2:**
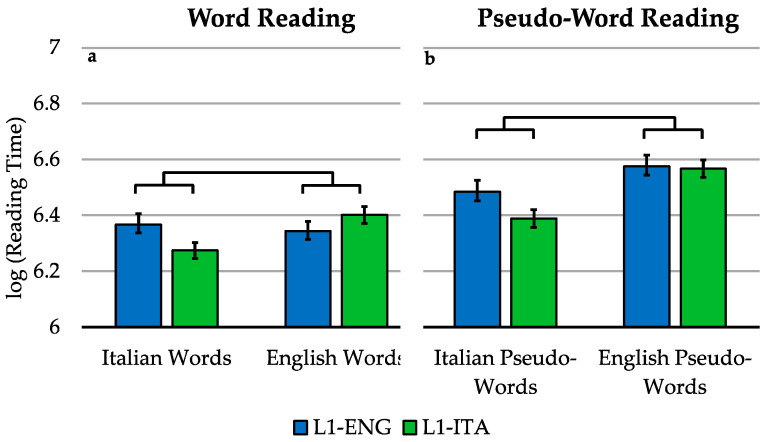
Word reading and pseudo-word reading. Mean log-transformed voice onset times for the reading task in the two languages for L1-Italian and L1-English bilinguals. Panel (**a**) refers to word reading. Panel (**b**) refers to pseudo-word reading. Error bars indicate the standard error of the mean. The reading times refer to accurate trials.

The 2 (group) by 2 (language) model (see Figure 2a) revealed that, overall, Italian words were read significantly faster than English words [F(1, 76.17) = 19.622, *p* < 0.001], whereas the main effect of the group was not significant [F(1, 37.98) = 0.176, *p* = 0.677]. We found a significant language-by-group interaction [F(1, 2885.5) = 255.654, *p* < 0.001]. The planned comparison revealed that the difference in reading times between L1-Italian and L1-English bilinguals is bigger with Italian words than with English words [χ2(1) = 255.65, *p* < 0.001].

### 3.3. Pseudo-Word Reading (in Blocks)

For pseudo-word reading, another group-by-language model was fitted (Figure 2b). We found that, overall, Italian pseudo-words were read significantly faster than English pseudo-words [F(1, 78.17) = 70.254, *p* < 0.001], while the main effect of the group was not significant [F(1, 38) = 1.225, *p* = 0.275]. Crucially, we also observed a significant language-by-group interaction [F(1, 2936.53) = 56.795, *p* < 0.001]. The planned comparison revealed that the difference in reading times between L1-Italian and L1-English bilinguals is bigger with Italian pseudo-words than with English pseudo-words [χ^2^(1) = 56.795, *p* < 0.001].

### 3.4. Word Reading (Mixed Presentation)

We assessed whether the reading speed advantage seen in the Italian bilinguals could be explained by their ability to access to their first orthography (O1) representation more efficiently. This time, the Italian and English stimuli were mixed and presented in a randomized order (e.g., table, parco, oven, apple, turtle, erba, etc.). These results were then compared with those collected using a block design. Differently from reading Italian or English stimuli presented in blocks, this task implies code switching. Previous literature on written language suggests that the processing costs of a O1-to-O2 switch could differ from those of a O2-to-O1 switch [44,45,46]. However, this account does not assume that access to the decoding strategy of either language could differ in terms of orthographic regularity and order of learning of the two orthographies. Accordingly, if the two orthographies can be used as efficiently with an efficient, segregated access by the two groups of bilinguals, only a main effect of first–second orthography is anticipated. Conversely, any orthography by group interaction for the effect of task would speak in favor of our initial hypothesis advocating that the effect of first orthographic exposure may have a different impact on reading stimuli from two languages characterized by a different degree of transparency.

**Figure 3:**
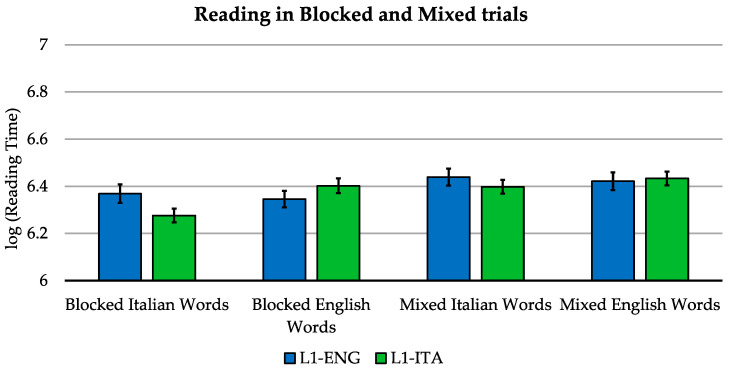
Reading in blocked and mixed trials: mean log-transformed voice onset times for block and mixed trials in the two languages for L1-Italian and L1-English bilinguals. Error bars indicate the standard error of the mean. The reading times refer to accurate trials.

The analysis was carried out using a 2 (language) by 2 (task: blocked vs. mixed) by 2 (group) model (see Figure 3). We replicated the main effect of language [F(1, 134.3)v = 11.614, *p* = 0.001] with the Italian stimuli being read faster, as well as the language-by-group interaction [F(1, 5094.4) = 206.331, *p* < 0.001]. In addition, we found a main effect of task [F(1, 134.5) = 67.595, *p* < 0.001] with blocked trials being associated with faster responses than for mixed trials, a language-by-task interaction [F(1, 134.4) = 5.516, *p* = 0.020], and a language-by-task-by-group interaction [F(1, 5094.2) = 47.395, *p* < 0.001]. The group-by-task interaction was not significant [F(1, 5094.8) = 0.371, *p* = 0.542].

**Figure 4:**
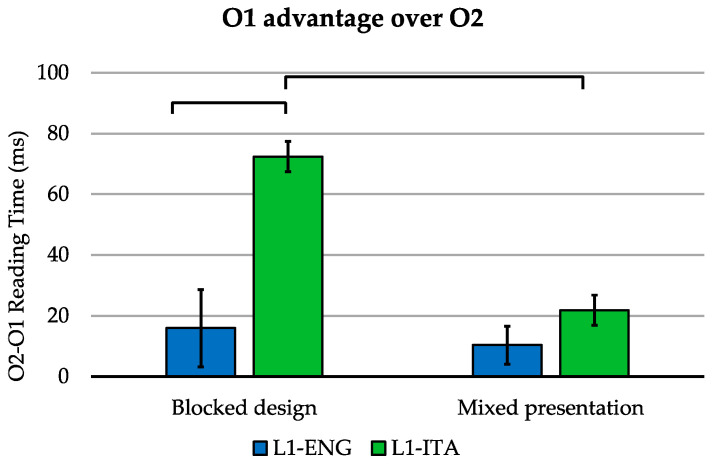
Reading time advantage for O1 over O2 in blocked and mixed reading tasks (accurate trials) for L1-Italian and L1-English bilinguals. Error bars indicate the standard error of the mean.

The language-by-task-by-group interaction effect (Figure 4) was further explored as follows: for each group, we computed (separately for the two tasks) the advantage of O1 over second orthography (O2), in terms of the difference in voice onset reading times between O2 and O1. Accordingly, positive values indicate an advantage for O1 stimuli. The data were further assessed by means of non-parametric pairwise comparisons.

We found that that L1-Italian bilinguals had a significantly greater O1 advantage than L1-English bilinguals in the blocked design [W = 65, *p* < 0.001], while no such difference emerged for the randomized presentation of Italian and English stimuli [W = 197, *p* = 1.000]. Remarkably, while L1-Italian bilinguals showed a significantly greater O1 advantage in the blocked design compared to the randomized presentation [V = 163, *p* < 0.001], this effect was not significant in L1-English bilinguals [V = 129, *p* = 1.000].

It is worth noting that the aforementioned significant interactions were present even when word frequency, bigram frequency, orthographic neighborhood size (N-size) and the initial phoneme were included in the models as covariates, as revealed by control analyses (see Supplementary Materials).

Alternative hypotheses—e.g., differential task switching costs effects—did not hold true either as shown with additional control tasks (see Supplementary Materials, Figure S3).

### 3.5. Interaction between L1 and L2 during Reading Acquisition: Computer Simulations

Our computer simulations were based on the connectionist dual process (CDP++) model of reading aloud [19]. We used the most recent and developmentally plausible version of the CDP model [42] to assess whether the present pattern of results in bilinguals can be explained by the model’s learning mechanisms. It is important to highlight that the knowledge of (sub-lexical) spelling–sound mappings in CDP is not acquired in the form of explicit rules but is implicitly encoded in the weights of the connections that link graphemes and phonemes in a neural network trained with associative learning. Orthographic learning of words is bootstrapped by decoding through the spelling–sound mapping network, which allows access to phonological word forms [42].

We explored the interaction between L1 and L2 learning to read using a minimal, basic assumption: knowledge of the L1 spelling–sound mapping (encoded in the L1 decoding network weights) was transferred to the decoding network of the L2 model before starting the learning loop. In other words, we first trained English and Italian L1 models, and then used weights from these to initialize L2 models. More specifically, weights connecting graphemes to phonemes in the L1 model were transferred to an L2 model if there was the same grapheme and a similar phoneme in the L2 language. The L2 models were then trained. Testing involved presenting the same items (words and pseudowords) used in the human experiment and collecting naming latencies to each model (see Figure S4 and Supplementary Materials for details).

**Figure 5:**
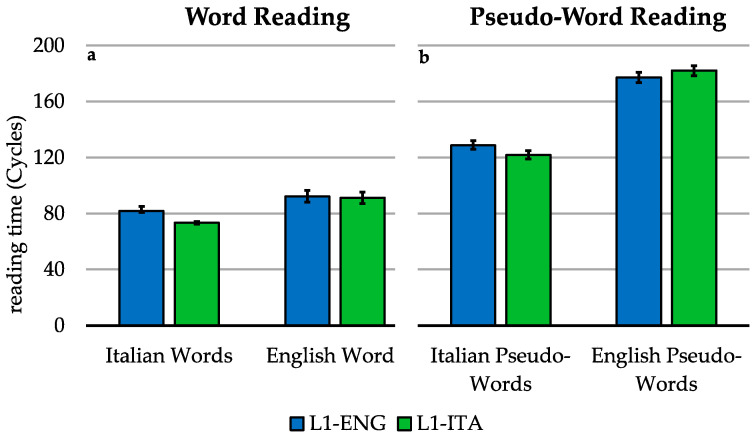
Reading performance of the computational reading models for words (**a**) and pseudo-words (**b**). The color of the bars identifies the L1 of each model. For example, a blue bar for Italian stimuli reflects the performance of a L2-Italian network (“equivalent” to an English bilingual). A green bar for English stimuli reflects the performance of an L2-English network (“corresponding” to an Italian bilingual). To match the human data, the L2-Italian network was trained with 150,000 word presentations; the other networks were trained with 300,000 presentations. See the main text for the caveats on the wording “corresponding”. Error bars indicate the standard error of the mean.

Our simulations (Figure 5) produced a pattern of results comparable to the human data: for Italian stimuli, the L1 network (“equivalent” to Italian bilinguals reading Italian in the behavioral study) was faster than the L2 network (“equivalent” to English bilinguals reading Italian); as for the human data, with English stimuli, the L1 and L2 networks performed more similarly (see Figure 5). For pseudowords, this was a stable feature in the simulations, in agreement with the hypothesis that the effect is driven by the consistency of sub-lexical spelling–sound associations.

Capturing the difference for real words required the additional assumption that the English L2 model (simulating an Italian subject learning English) had to be trained longer than the Italian L2 model (simulating an English subject learning Italian). This assumption reflects the fact that reaching a high level of proficiency in reading English requires longer training, in comparison to Italian. Moreover, even though the number of years of L2 reading was balanced across the bilinguals, Italian bilinguals began learning the second orthography earlier than the English bilinguals (cf. Table 1), thereby providing further support to the aforementioned assumptions for computer simulations.

Analyses of the connection weights of the models highlighted important differences between models (see the Supplementary Materials for details). Notably, the number of weights in the models transferred from L1 to L2 (which indicates the amount of grapheme–phoneme mapping knowledge coming from L1) was very similar from L1 Italian to L2 English and from L1 English to L2 Italian. However, after learning L2, the number of weights finally used varied dramatically, with many more weights used in English than Italian. This means that the proportion of weights that would have been affected by the weight transfer is much greater in the L2 Italian (“equivalent” to English who learn Italian) than the L2 English network (equivalent to Italians who learn English).

This computational evidence suggests that a greater amount of information needs to be learnt by Italian bilinguals when learning English because even though they start off with a similar amount of L2 transfer, there is simply a lot more that needs to be learnt in English. Furthermore, because English has an inconsistent orthography, the transfer of English weights to Italian transfers a lot of relationships not used in Italian which then need to be inhibited, whereas the transfer of weights from Italian to English is often but not always useful for English. This is another factor that would cause the effect of seeding the Italian L2 network to be more than the seeding of the English L2 network. It follows that there is proportionately less interference from the L1 to L2 when learning English as a second orthography compared to when learning Italian as a second orthography. This caused the distribution of weights in the L1 and L2 English models to be more similar than the L1 and L2 Italian models and provides a simple and plausible explanation for why the performance of the L1 and L2 English networks appear similar, while the L1 and L2 Italian networks do not (see the Supplementary Materials for further analyses).

Indeed, there were processing time differences for the L1 and L2 Italian models but not for the English models. This pattern mirrors what is seen in the two groups of bilinguals for reading Italian rather than English stimuli.

As noted above, the simulation assumed longer training for the English L2 model. When considering L1 and L2 models receiving the same amount of training (see Supplementary Materials for details), the advantage of the L1 Italian model over the L2 Italian model did not reach a corrected significance level, whereas the effect remained significant for non-words (see Figure S5). This suggests that with indefinitely long training, English bilinguals may catch up with Italian bilinguals in terms of performance on known Italian words. Nevertheless, they would still process novel Italian orthographic strings of pseudo-words less proficiently than L1-Italian subjects.

## 4. Discussion

The implications of these experiments are simple: as described for auditory phonological discrimination, high-proficiency bilingual readers maintain signs of the order with which their two orthographies were acquired, at least when the two orthographies differ in terms of consistency. This is demonstrated by the fact that the reading performance of our bilinguals did not show simple L1 versus L2 effects; instead, the behavioral patterns showed a significant interaction between the consistency of the orthography and the order of its acquisition: the advantage for the L1-Italian bilinguals when reading Italian was greater than that of L1-English bilinguals when reading English. These results could not be explained by other potential confounding factors, such as differences in baseline cognitive abilities or executive control. The two groups of bilinguals were well matched for elementary visuo-vocal reaction times, articulation speed and ease of word retrieval (as measured by the semantic fluency task); they were also matched on executive control (see Supplementary Materials).

Remarkably, our data show an important departure from what described for oral language processing: Cutler et al. [16,17] concluded that people process oral language only with the phonological representations and segmentation routines of their first language. Whilst this position is not ubiquitous (see, for example, [18]), it provides a good starting point of discussion. The question here was: do bilingual readers process orthographic strings with a unitary mechanism, or do they utilize segregated orthography-specific mechanisms? Our findings in high-proficiency bilinguals suggest that (1) the mechanisms may not be unitary for all bilinguals and (2) one important factor is the consistency of the orthographies to be learned and the order of the learning. Our data suggest that learning a relatively consistent orthography first may be beneficial because it allows the fast learning and hence early recruitment of efficient sets of orthographic representations for reading words or pseudo-words when needed.

The fact that performance between L1-Italian and L1-English bilinguals was more similar while reading English stimuli than while reading Italian stimuli also suggests that the L1-Italian bilingual readers were more efficient than L1-English bilinguals in accessing language-specific grapheme–phoneme representations. Importantly, L1-Italian readers may also benefit from the fact that their sub-lexical grapheme-to-phoneme computations never clash with the phonology, indicated by representations of a larger size when reading Italian stimuli (for a discussion, see [30]).

On the other hand, the English stimuli appear to possess unsurmountable constraints whereby their processing cannot be further improved whatever the additional reading training, such as that implied by learning a regular orthography: it is telling that for English pseudo-words, where no lexical familiarity can play a major role, the reading times were virtually identical for both groups of bilinguals.

Our data also suggest that the learning of an more inconsistent orthography first does not allow one to catch up fully with the simplicity of a second, more consistent orthography: while learning occurred in L1-English bilinguals, as shown by the errorless performance with both Italian pseudo-words and words, overall, they did not seem to benefit as much from the simplicity of the more consistent orthography, at least in terms of decoding speed: this was comparatively less that for the Italian bilinguals. We suggest that English bilinguals might be using a broader and less segregated set of orthographic representations at all times, and that the orthography-to-phonology inconsistencies of their O1 may negatively affect the acquisition and/or use of the correspondences of a consistent O2. The latter hypothesis was supported by the simulations with the CDP++ computational model of reading aloud. Starting from the simple assumption that knowledge of the O1 spelling–sound mapping (i.e., the network connectivity) acts as a prior for learning the O2 orthography, we observed an interaction between orthographies that was modulated by the consistency of the mapping. In particular, seeding associative learning with previous knowledge about the English spelling-mapping was of little help for learning to read Italian because the inconsistency of English is expressed by an intricate pattern of associations (i.e., connection weights), with many unusual print-to-sound correspondences that need to be inhibited in the course of learning.

Our computational simulations clearly speak in favor of an order effect from the learning of a more regular or a more irregular orthography: however, the model, for the time being, cannot mimic a bilingual reader fully. It therefore cannot tell, for example, to what extent the mappings of two orthographies are kept segregated at a functional level. Nevertheless, our behavioral data in which we compared a blocked versus mixed presentation of the stimuli of two orthographies provide hints on this matter: a significant advantage for the block-design presentation was present only in the Italian readers, while the English readers did not benefit from the blocked presentation, with comparable and only marginal advantages for O1 stimuli in both the blocked and randomized version of the reading task. This suggests that for L1-Italian bilinguals, the two sets of orthographic representations are sufficiently separated at a functional level to such an extent that, when one such bilingual reads Italian writing in a blocked manner—e.g., when reading an Italian novel, or a newspaper—the “Italian” codes have privileged and highly efficient access. At present, we cannot tell whether this may depend on the existence of two fully separated spelling–sound mechanisms and orthographic lexicons—one for Italian and one for English—or whether the two are just sufficiently well segregated at a functional level to permit, under certain circumstances, a privileged access to one of the two ends of the same reservoir of spelling–sound correspondences. At a functional level, the two hypotheses provide similar explanations on the mechanism whereby the L1-Italian readers should pay a price while reading Italian words mixed with English words: we propose that this should arise from a specific switching cost from two at least partially segregated pools of spelling–sound representations. In the same vein, we can postulate that for L1-English bilinguals, no such cost is paid because they operate equally slowly when reading in random order due to the larger and more shared pool of correspondences.

It is worth noting that stimuli were not perfectly balanced in terms of frequency, bigram frequency and orthographic neighborhood size (see Supplementary Materials, Table S7). Although the lack of fully comparable Italian and English frequency corpora limits the extent to which these differences could be attributed to structural properties of the two languages, it could be pointed out that our findings could be due to Italian stimuli being “easier” to process than English stimuli. Accordingly, independent samples Mann–Whitney tests indicate significant language effects whereby, overall, Italian stimuli are significantly more frequent, have a higher bigram frequency and a greater orthographic neighborhood than the English ones. However, if reading was only influenced by cross-linguistic differences in stimuli (with Italian stimuli easier to read than the English ones), then L1-Italian and L1-English bilinguals should have performed equally well with Italian stimuli and equally badly with English stimuli. As the results indicate, while for English stimuli the two groups performed similarly, for the Italian ones, there was a solid advantage of L1-Italian bilinguals over L1-English bilinguals: this cannot be explained in terms of a main effect of language. In our view, this finding suggests that, regardless of the overall “simplicity” of the stimuli that have to be read, a reader whose orthographic decoding system was first trained with a more opaque orthography cannot fully catch up with the efficiency of the reading system first trained to decode a more transparent orthography.

Our data complement a recent proposal in bilingual research: assuming that language-specific grapheme-to-phoneme mapping rules shape bilingual lexical organization [26], bilingualism may lead to an “accommodation”, a blending of the reading strategies typical of the orthographies the bilinguals are exposed to [29]. However, while such accommodation is likely to occur in early acquisition bilinguals who learn two orthographies at the same time, for late-acquisition bilinguals (although with extremely high-proficiency in both languages), this might not be the case to the same extent. Indeed, our data suggest that bilingual reading may vary in terms of segregation of the language-specific representations depending on the regularity of the learned orthographies and the order of such learning. In other words, we anticipate that orthographic “accommodation” effects and order effects (such as those described in this work) could represent the extremes of a continuum of possible outcomes of bilingual reading, with the position on this continuum being determined by the difference in terms of plasticity of the neurocognitive system at the moment of first- and second-orthography acquisition. In other words, the smaller the difference in the plasticity of the neurocognitive system at the moment of first and second orthography acquisition, the more we would expect the “accommodation” phenomenon to occur; conversely, the greater the difference in plasticity, the more we would expect to observe an “order” effect. In any event, although the available evidence from early acquisition Italian-English and English-Italian bilinguals is not incompatible with this hypothesis (see, for instance, [47,48]), further studies will be necessary to test its predictive value.

These results raise a number of questions that remain in search of answers from future experiments. Would the differences in decoding times also translate into a less efficient performance in reading comprehension tasks? Would the situation demonstrated for these groups also apply to early exposure bilinguals of comparable proficiency? There is one prediction that one could make, namely that no such differences should be apparent —or if anything, they should be much smaller—provided that the bilingualism is for languages that both have a transparent orthography with similar conversion rules, and all other factors are kept balanced as they were here. Finally, it is worth considering that Italian and English orthographies share the same orthographic symbols (Latin letters). Future studies will also need to address the extent to which the conclusions of the present work apply also to bilingualism for languages with different writing systems, such as, for example, Italian (or English) and Chinese (see, for instance, [49]). Research in this direction, we believe, will also provide insightful information about the core mechanisms that determine cross-linguistic transfer of print-to-sound mappings.

## 5. Conclusions

To conclude, our findings demonstrate orthographic constraints on bilingual reading, whereby the level of consistency of the first learned orthography affects later learning and performance on a second orthography. It is interesting that the novel computer simulations presented here were consistent with the conclusions derived from human data, providing a further validation to our findings. Taken together, these observations set the rationale for similar investigations in different age groups or in subjects with a developmental reading disorder to identify the best timing for the learning of a second orthography depending on its regularity and the baseline reading skills of the learner. The same findings also set the rationale for imaging brain investigations on whether the subtle differences observed in the two groups of bilinguals could be characterized in neurofunctional terms as well.

## Figures and Tables

**Table 1:** Demographic data and information on bilingual experience of the participants: age and education (years); handedness (evaluated using the Oldfield inventory); age of onset of acquisition of first and second language (spoken; years); percentage of daily oral and written exposure to either Italian or English (based on self-reports made by the participants); experience with second orthography (O2, years); vocal reaction times to a simple visual stimulus (in milliseconds [ms]); articulation speed (number of repetitions of a target pair of words in 15 s); number of correct Italian-to-English and English-to-Italian translations (30 words of high, intermediate, and low frequency for either language as in [35]). For further details, see the Materials and Methods section and Supplementary Materials.

	Group	Age (Years)	Education (Years)	Handedness (Oldfield Inventory)	Age of onset of L1 Acquisition (Years)	Age of onset of L2 Acquisition (Years)	% Daily Exposure to Italian (Oral)	% Daily Exposure to English (Oral)
mean	L1-English	42.364	16.955	0.925	0.545	12.000	63.182	36.591
	L1-Italian	34.611	16.667	0.863	0.417	7.250	72.778	27.222
sd	L1-English	11.701	1.704	0.112	1.99	9.730	21.742	21.843
	L1-Italian	14.561	1.534	0.144	0.974	5.056	13.198	13.198
N	L1-English	22	22	22	22	22	22	22
	L1-Italian	18	18	18	18	18	18	18
Statistic (df)		t(38) = 1.868	U = 172	U = 131.500	U = 185.500	U = 140	U = 146	U = 148.500
*p* value		0.069	0.454	0.063	0.570	0.114	0.154	0.176
	**Group**	**% of Daily Exposure to Italian (Written)**	**% of Daily Exposure to English (Written)**	**Experience with O2 (years)**	**Vocal Reaction Time (ms)**	**Articulation Speed (Number of Repetitions)**	**Italian-to-English Translations (Number of Correct Translations)**	**English-to-Italian Translations (Number of Correct Translations)**
mean	L1-English	39.727	60.045	26.682	329.386	23.773	27.409	29.500
	L1-Italian	56.111	43.889	25.528	346.083	25.056	25.389	29.389
sd	L1-English	26.057	25.852	12.506	50.122	4.140	2.520	1.012
	L1-Italian	21.182	21.182	14.529	67.121	4.022	3.567	1.145
N	L1-English	22	22	22	22	22	22	22
	L1-Italian	18	18	18	18	18	18	18
Statistic (df)		t(38) = −2.148	t(38) = 2.129	t(38) = 0.270	t(41) = −0.900	t(38) = −0.998	U = 133	U = 189
*p* value		0.038	0.040	0.789	0.374	0.330	0.075	0.761

**Table 2:** Stimuli, frequency, bigram frequency and orthographic neighborhood size. Mean frequency for words and bigram frequency as well as orthographic neighborhood size (N-size) for words and pseudo-words in the two languages according to the SUBTLEX-UK [36] and the SUBTLEX-IT [37].

Type	Task		Language	Type Summed BF	Token Summed BF (per Million)	Frequency	Frequency per Million	Zipf	N-Size	Letters
WORD	BLOCKED	mean	ITA	55,662.45	108,465.08	7116.75	76.52	4.65	22.28	4.75
		sd		25,174.08	46,889.87	9136.32	98.23	0.43	11.11	0.63
PSEUDO	BLOCKED	mean	ITA	48,560.33	89,805.72				15.73	4.58
		sd		21,840.57	39,337.13				10.84	0.59
WORD	MIXED	mean	ITA	56,408.17	112,847.74	10,556.10	113.50	4.50	22.83	4.73
		sd		21,624.58	39,588.68	24,510.49	263.54	0.67	13.47	0.64
WORD	BLOCKED	mean	ENG	28,662.43	81,156.41	6164.70	30.62	4.23	8.08	5.60
		sd		10,143.13	36,062.04	6879.64	34.17	0.50	7.01	0.67
PSEUDO	BLOCKED	mean	ENG	26,376.03	73,593.46				6.53	5.55
		sd		11,255.54	30,752.87				6.56	0.60
WORD	MIXED	mean	ENG	30,477.97	86,276.20	4435.23	22.03	4.11	6.03	5.80
		sd		8148.82	27,106.47	6300.77	31.29	0.44	3.64	0.41

**Table 3:** Descriptive statistics for the reading tasks in the two groups.

Lexicality	Words					Pseudo-Words	
**Presentation Type**		**Blocked**		**Mixed**		**Blocked**	
Language		Italian	English	Italian	English	Italian	English
mean	L1-English	592.853	576.94	634.218	623.858	667.613	728.634
	L1-Italian	535.653	608.017	605.056	626.870	599.884	717.023
sd	L1-English	106.246	88.619	101.709	103.030	129.005	128.013
	L1-Italian	63.831	77.310	72.953	75.173	77.178	93.824

## Data Availability

The data presented in this study are available on https://osf.io/86c9v/.

## Supplementary Materials

The following are available online at https://www.mdpi.com/article/10.3390/brainsci11070878/s1, Figure S1: Distribution of the weights in Italian and English CDP++ monolingual models (see introduction in the manuscript), Figure S2: Distribution of behavioral data, Supplementary methods and results, Table S1: results (ANOVA) of control analyses for the blocked word reading task, Table S2: results (ANOVA) of control analyses for the blocked pseudo-word reading task, Table S3: results (ANOVA) of control analyses for blocked/randomized word reading, Figure S3: Stroop Effect, Figure S4: Reaction times and error rates of the models across 500,000 word presentations, Figure S5: Results of the models after training the L1 and L2 networks on 300,000 word presentations, Table S4: Frequency data for the proficiency test, Table S5: Experimental Stimuli, Table S6: Normality tests for the control tasks, Table S7: Cross-linguistic comparison of psycholinguistic data for experimental stimuli, Table S8: %accurate trials in all experimental conditions, Section: grapheme–phoneme correspondences used to seed the L2 networks.
