# Supplementary material for: Effects of Orthographic Consistency on Bilingual Reading: Human and Computer Simulation Data
Source: Brain Sci. 2021 Jun 30;11(7):878. doi: 10.3390/brainsci11070878 (PMC8301906; doi:10.3390/brainsci11070878)
Supplement: Supplementary file 1 [file brainsci-11-00878-s001.zip › brainsci-1237419-supplementary.pdf]

Article

Effects of Orthographic Consistency on Bilingual Reading: Human and Computer Simulation Data

Eraldo Paulesu [1,2,†], Rolando Bonandrini [1,*,†], Laura Zapparoli [1], Cristina Rupani [1], Cristina Mapelli [3], Fulvia Tassini [4], Pietro Schenone [4], Gabriella Bottini [5,6], Conrad Perry [7] and Marco Zorzi [8,9,*]

1 Psychology Department, University of Milano- Bicocca, Milano, Italy
2 IRCCS Istituto Ortopedico Galeazzi, Milano, Italy
3 School of Medicine and Surgery, University of Milano-Bicocca, Milano, Italy;
4 Civica Scuola Interpreti e Traduttori, Milano, Italy
5 Department of Brain and Behavioural Sciences, University of Pavia, Pavia, Italy
6 Cognitive Neuropsychology Centre, ASST "Grande Ospedale Metropolitano" Niguarda, Milan, Italy
7 Department of Psychology, The University of Adelaide, Adelaide, Australia
8 Department of General Psychology and Padova Neuroscience Centre, University of Padova, Padova, Italy
9 IRCCS Ospedale San Camillo, Venice-Lido, Italy
* Correspondence: r.bonandrini@campus.unimib.it (R.B.); marco.zorzi@unipd.it (M.Z.)
† Both authors equally contributed to this work.

Supplementary Material

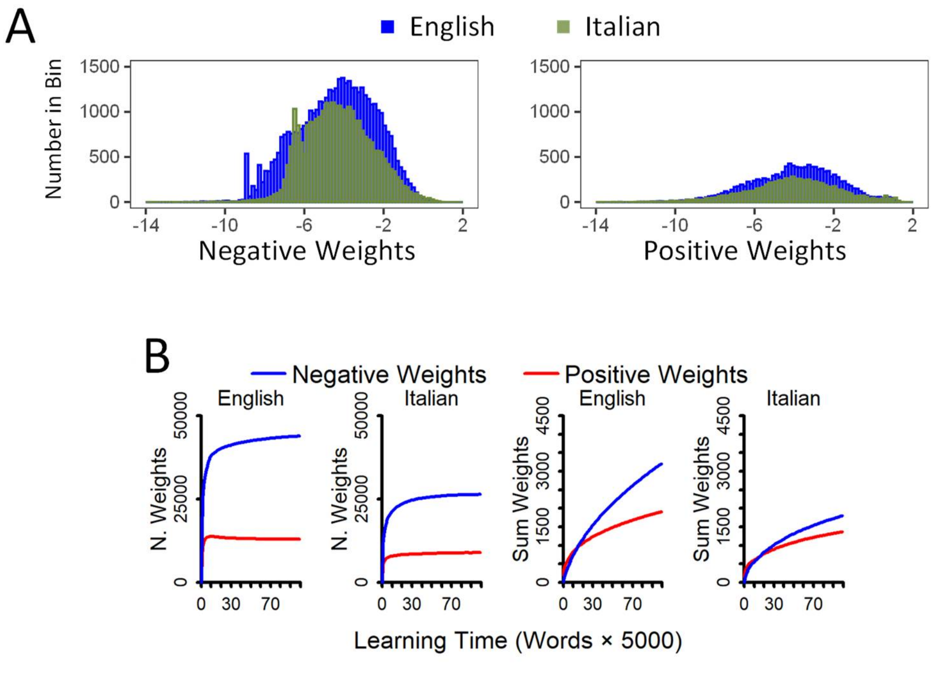

Figure S1: Distribution of the weights in Italian and English CDP++ monolingual models (see introduction in the manuscript). Distribution of weights describing the connectivity between graphemes and phonemes in the associative neural network that is part of the CDP++ model of reading aloud, in its English and Italian monolingual versions [1, 2] The negative and positive weights are represented separately because they have different distributions and their purpose is different. Positive weights cause phonemes to be activated and increase in size whenever a grapheme and a phoneme co-occur together in training. Negative weights inhibit the phoneme of the grapheme they are connected to; they also increase in size when a phoneme is predicted from a given word but is not actually in the word. Panel A shows the distribution of log weight values for the two monolingual networks after 300,000 word presentations. Panel B shows how the number of non-zero weights and the total sum of the weights develops during learning in the two monolingual networks.

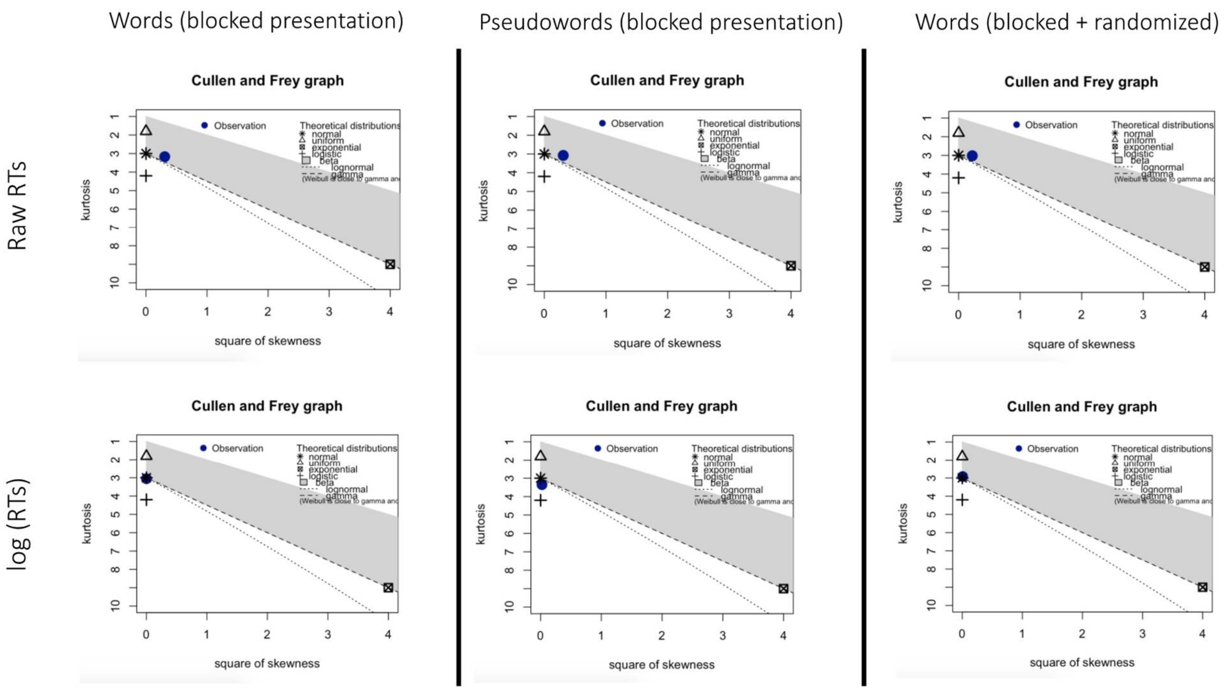

Figure S2: Distribution of behavioural data. Cullen and Frey graphs indicating distribution of behavioural data in the three analyses. The top quadrants show distribution of raw RTs, while the bottom quadrants show the distribution of log-transformed RTs.

Supplementary Methods and Results

Behavioural data

Description of the Proficiency Test: Subjects were asked to translate words of different frequency (high, intermediate, low) from English to Italian (30 words each) and vice versa. For each subject, the number of correct translations for each language was computed.

Control analyses

We performed a series of control analyses, in which different covariates were inserted in the models with the aim of excluding that the interactions of interest (namely the Language-by-Group interaction in the blocked tasks and the Language-by-Group-by-Task interaction in the randomized reading task) could be cancelled-out after controlling for more general orthographic and phonemic phenomena. For what concerns lexical stimuli, word frequency (Zipf values [3]) was computed. In addition, in order to control any possible effect of word onset on vocal reaction times [4], the initial phoneme of each Italian and English word (encoded in the International Phonetic Alphabet) was derived from wikitionary (https://en.wiktionary.org/wiki/Wiktionary:Main_Page; the website https://tophonetics.com was also used for one English stimulus and the website https://www.dizionario-italiano.it/ for one Italian word).

For what concerns both words and pseudo-words, measures of bigram frequency and orthographic neighbourhood were computed. As far as bigram frequencies are concerned, we computed position-independent Summed Bigram Frequency (SBF) for each stimulus.

More precisely, two metrics of bigram frequencies were computed: Type SBF and Token SBF (frequency per million). The first one counts the occurrence of a bigram in the set of words present in a corpus (without multiplying for frequency). Multiple occurrences of a target bigram in the same words are taken into account. The second index, instead, weights the bigram frequency count for the word frequency (per million) of the words in which the bigram is found.
As far as orthographic neighbourhood (N) size [5] is concerned, we calculated (substitution) N size for the two SUBTLEX corpora. In particular, for the SUBTLEX-IT, letters marked by orthographic stress were substituted by the corresponding capital letters and items with frequency =1 were eliminated, as well as non-alphabetic entries. Subsequently, for each target item, the number of words in the corpus with the same length which were equal to the target stimulus minus one letter (N size) was computed. In order to make the computation of N size comparable in the two languages, we did so also for English stimuli with reference to the SUBTLEX-UK.

Blocked presentation of Italian and English words

In these control analyses, none of the covariates showed a significant effect (Table S1). Remarkably, in none of the models the crucial interaction effect became non-significant after the inclusion of the covariate.

Table S1: results (ANOVA) of control analyses for the blocked word reading task. The symbol ":" indicates interaction effects.

		Sum Sq	Mean Sq	NumDF	DenDF	F value	Pr(>F)
original model	Language	0.322	0.322	1.000	76.170	19.622	< 0.001
	Group	0.003	0.003	1.000	37.980	0.177	0.677
	Language:Group	4.193	4.193	1.000	2885.500	255.654	< 0.001
control for initial phoneme	Phoneme	0.501	0.026	19.000	56.750	1.606	0.086
	Language	0.318	0.318	1.000	57.220	19.417	< 0.001
	Group	0.003	0.003	1.000	37.980	0.176	0.677
	Language:Group	4.191	4.191	1.000	2885.270	255.529	< 0.001
control for frequency (Zipf)	Zipf	0.001	0.001	1.000	75.150	0.051	0.821
	Language	0.275	0.275	1.000	74.920	16.771	< 0.001
	Group	0.003	0.003	1.000	37.980	0.177	0.677
	Language:Group	4.193	4.193	1.000	2885.400	255.639	< 0.001
control for type Bigram Frequency	type_BF	0.014	0.014	1.000	75.070	0.853	0.359
	Language	0.281	0.281	1.000	75.200	17.139	< 0.001
	Group	0.003	0.003	1.000	37.980	0.177	0.677
	Language:Group	4.192	4.192	1.000	2885.570	255.633	< 0.001
control for token Bigram Frequency (frequency per million)	token_BF_perM	0.037	0.037	1.000	74.760	2.243	0.138
	Language	0.364	0.364	1.000	75.110	22.170	< 0.001
	Group	0.003	0.003	1.000	37.980	0.176	0.677

	Language:Group	4.191	4.191	1.000	2885.560	255.530	< 0.001
control for orthographic neighbourhood (N) size	N	0.025	0.025	1.000	74.470	1.502	0.224
	Language	0.126	0.126	1.000	74.760	7.671	0.007
	Group	0.003	0.003	1.000	37.990	0.177	0.677
	Language:Group	4.192	4.192	1.000	2885.450	255.580	< 0.001

Blocked presentation of Italian and English pseudo-words

In these control analyses, only the effect of N size turned out to be significant. Also in these analyses, in none of the models the crucial interaction effect became non-significant after the inclusion of the covariate.

Table S2: results (ANOVA) of control analyses for the blocked pseudo-word reading task. The symbol ":" indicates interaction effects.

		Sum Sq	Mean Sq	NumDF	DenDF	F value	Pr(>F)
original model	Language	1.669	1.669	1.000	78.170	70.254	< 0.001
	Group	0.029	0.029	1.000	38.000	1.226	0.275
	Language:Group	1.349	1.349	1.000	2936.530	56.795	< 0.001
control for type Bigram Frequency	type_BF	0.042	0.042	1.000	77.040	1.770	0.187
	Language	1.442	1.442	1.000	77.330	60.725	< 0.001
	Group	0.029	0.029	1.000	38.000	1.227	0.275
	Language:Group	1.350	1.350	1.000	2936.570	56.839	< 0.001
control for token Bigram Frequency (frequency per million)	token_BF_perM	0.006	0.006	1.000	216.800	0.269	0.605
	Language	0.590	0.590	1.000	217.200	26.725	< 0.001
	Group	0.011	0.011	1.000	38.000	0.510	0.479
	Language:Group	5.266	5.266	1.000	8128.000	238.684	< 0.001
control for orthographic neighbourhood (N) size	N	0.163	0.163	1.000	216.700	7.376	0.007
	Language	0.215	0.215	1.000	217.000	9.727	0.002
	Group	0.011	0.011	1.000	38.000	0.510	0.480
	Language:Group	5.265	5.265	1.000	8128.000	238.649	< 0.001

Blocked and mixed presentation of Italian and English words

In these control analyses, only the effect of initial Phoneme turned out to be significant. It is worthy to note that, after controlling for N size, the main effect of Language was no longer significant. However, in none of these models the crucial interaction effect became non-significant after the inclusion of the covariate.

Table S3: results (ANOVA) of control analyses for blocked/randomized word reading. The symbol ":" indicates interaction effects.

		Sum Sq	Mean Sq	NumDF	DenDF	F value	Pr(>F)
original model	Language	0.189	0.189	1.000	134.400	11.614	< 0.001
	Group	0.002	0.002	1.000	38.000	0.148	0.702
	Task	1.101	1.101	1.000	134.500	67.595	< 0.001
	Language:Group	3.360	3.360	1.000	5094.400	206.331	< 0.001
	Language:Task	0.090	0.090	1.000	134.400	5.516	0.020
	Group:Task	0.006	0.006	1.000	5094.800	0.371	0.542
	Language:Group:Task	0.772	0.772	1.000	5094.200	47.395	< 0.001
control for initial phoneme	Phoneme	1.002	0.044	23.000	110.300	2.676	< 0.001
	Language	0.231	0.231	1.000	110.700	14.203	< 0.001
	Group	0.002	0.002	1.000	38.000	0.147	0.703
	Task	1.337	1.337	1.000	110.700	82.074	0.000
	Language:Group	3.352	3.352	1.000	5094.400	205.860	< 0.001
	Language:Task	0.074	0.074	1.000	110.700	4.527	0.036
	Group:Task	0.006	0.006	1.000	5094.600	0.370	0.543
	Language:Group:Task	0.770	0.770	1.000	5094.400	47.286	< 0.001
control for frequency (Zipf)	Zipf	0.020	0.020	1.000	132.600	1.228	0.270
	Language	0.124	0.124	1.000	133.100	7.589	0.007
	Group	0.002	0.002	1.000	38.000	0.148	0.702
	Task	1.046	1.046	1.000	133.400	64.261	< 0.001
	Language:Group	3.360	3.360	1.000	5094.300	206.364	< 0.001
	Language:Task	0.089	0.089	1.000	133.300	5.449	0.021
	Group:Task	0.006	0.006	1.000	5094.700	0.368	0.544
	Language:Group:Task	0.772	0.772	1.000	5094.100	47.433	< 0.001
control for type Bigram Frequency	type_BF	0.000	0.000	1.000	133.000	0.003	0.957
	Language	0.119	0.119	1.000	133.300	7.296	0.008
	Group	0.002	0.002	1.000	38.000	0.149	0.702
	Task	1.092	1.092	1.000	133.500	67.035	< 0.001
	Language:Group	3.360	3.360	1.000	5094.300	206.338	< 0.001
	Language:Task	0.089	0.089	1.000	133.400	5.471	0.021
	Group:Task	0.006	0.006	1.000	5094.800	0.371	0.542
	Language:Group:Task	0.772	0.772	1.000	5094.200	47.399	< 0.001
control for token Bigram Frequency (frequency per million)	token_BF_perM	0.007	0.007	1.000	132.600	0.430	0.513
	Language	0.191	0.191	1.000	133.300	11.742	< 0.001
	Group	0.002	0.002	1.000	38.000	0.148	0.702
	Task	1.081	1.081	1.000	133.500	66.383	< 0.001
	Language:Group	3.359	3.359	1.000	5094.300	206.306	< 0.001
	Language:Task	0.090	0.090	1.000	133.400	5.506	0.020

	Group:Task	0.006	0.006	1.000	5094.800	0.372	0.542
	Language:Group:Task	0.772	0.772	1.000	5094.200	47.393	< 0.001
control for orthographic neighbourhood (N) size	N	0.045	0.045	1.000	132.800	2.743	0.100
	Language	0.043	0.043	1.000	132.800	2.608	0.109
	Group	0.002	0.002	1.000	38.000	0.148	0.702
	Task	1.096	1.096	1.000	133.500	67.277	< 0.001
	Language:Group	3.358	3.358	1.000	5094.300	206.245	< 0.001
	Language:Task	0.099	0.099	1.000	133.300	6.100	0.015
	Group:Task	0.006	0.006	1.000	5094.800	0.373	0.541
	Language:Group:Task	0.772	0.772	1.000	5094.200	47.388	< 0.001

Tests of alternative interpretations of the blocked versus randomized presentation data.

The greater advantage for reading O1 stimuli in the block-design for the L1-Italian bilinguals suggests a very efficient reliance of an “Italian mode” of reading, when there is no uncertainty due to all of the following words being Italian in the block design. Hence, the Italian readers appear very efficient in isolating their sets of rules and the needed phonological retrieval mechanisms. On the other hand, the comparatively smaller vulnerability to the mixed presentation of the stimuli seen in the L1-English bilinguals suggests that they might use a broader set of orthographic representations whereby the continuing changes of orthography due to the mixed presentation has more limited costs for these readers.

Could this pattern be explained by a different, epiphenomenal, greater sensitivity in the L1-Italian bilinguals to the cognitive control demands implied by the random presentation of Italian or English words rather than to an orthographic effect? That is, a difference in their ability to suppress competing responses caused by a continuous switching across languages.

As much as this may appear unlikely, it seemed important to us to exclude this possibility. There are at least two arguments that militate against this cognitive control hypothesis: (1) the between-group difference for the performance in the randomized presentation of the stimuli was not significant: a greater overall vulnerability to task switching would anticipate a between-group difference in the mixed-presentation task; (2) the verbal fluency task, a task typically associated with strong prefrontal activations [6] depending on executive control [7], did not reveal a systematic superiority effect for the English group, at variance with what a cognitive control superiority hypothesis would predict.

Further evidence that the two groups were well balanced in terms of cognitive control and ability to suppress competing response comes from the **Stroop task**. If the English bilinguals had greater ability to suppress competing responses, then they should also show a smaller Stroop interference effect.

This test was used to assess whether the two groups were well balanced in terms of cognitive control and ability to suppress competing response, as needed during the mixed-language reading task. We reasoned that if one group of bilinguals had greater ability to suppress competing responses, then they should also show a smaller Stroop interference effect. We therefore administered our subjects with a computer version of the Stroop interference test [8]. Participants were asked to name the colour of the ink in which a target word like GREEN was printed. The colour of the word could either be congruent (e.g. RED) or incongruent (e.g. GREEN) with the meaning of the printed word. The difference between the vocal reaction time of the incongruent and congruent condition was taken as a measure of an interference suppression cost [9]. Participants were presented with two versions of the same task, one in Italian and one in English in a counterbalanced order among subjects.

Contrary to the prediction of a cognitive superiority effect for the L1 English group (see S2 Fig), we found a Stroop effect in both groups both for the Italian version [L1-Italian group $t(17) = 8.622$, $p < .001$, L1-English group $t(21) = 9.796$, $p < .001$, H0: mean = 0; H1: mean > 0] and the English version of the task [L1-Italian group $t(17) = 12.986$, $p < .001$, L1-English group $t(21) = 13.510$, $p < .001$, H0: mean = 0; H1: mean > 0].

Furthermore, an ANOVA model revealed that neither the main effects of Language [$F(1,38) = 2.985$, $p = .092$] and Group [$F(1,38) = 0.597$, $p = .445$], nor their interaction [$F(1.38) = 2.700$, $p = .109$] were significant. These results strongly suggest that the block versus random presentation effect described above cannot be explained in terms of different cognitive control abilities in the two groups with better such abilities in the L1-English subjects.

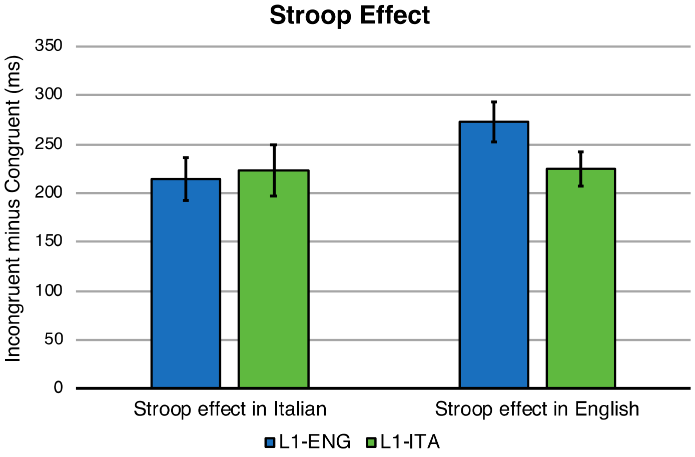

Figure S3: Stroop Effect. Mean Stroop effect in the two languages for L1-Italian and L1-English bilinguals. Error bars indicate Standard Error (SE).

Computational simulations of L2 learning

Learning to read was simulated using the most recent, developmental version, of the CDP++ model of reading aloud [10]. This is the only large-scale computational model that is capable of handling polysyllabic words (with a lexicon of tens of thousands of words) where both English and Italian language versions exist [1]. It allows written word representations to be learnt in its orthographic (written word) lexicon and relationships

between graphemes and phonemes to be learnt without whole-word (lexical) knowledge via a two-layer associative (TLA) network. Knowledge in these models is not explicitly encoded but is represented by the weights of the connections that link graphemes and phonemes in a neural network. Written word representations are typically learnt by giving words a chance of being lexicalized (i.e., added to the model's lexicon) if the model can decode the word into phonology using the TLA network and activate an entry in its phonological (spoken word) lexicon. A far less frequent way words are learnt is by giving the model the correct phonology. Being given the correct phonology is designed to represent teaching and other less automatic strategies that are used when decoding fails (e.g., [11]). It is simulated by giving each word that is not successfully decoded a small chance of being lexicalized. The model thus offers a developmental perspective on reading and allows performance to be examined at different time points in training.

The English model was identical to Perry et al. [10] except that rather than use a child lexicon, the standard adult database from [2] was used. The Italian language version had the same architecture and processing assumption of the English model, whereas language-specific features, the lexical database, and model parameters were the same as reported in [1] for the CDP++ Italian model. Because the Italian lexical database is approximately twice the size of the English database and we wanted to compare reaction times (RTs) to words and pseudo-words, two different rates of lexicalization were used. The Italian model had a 30% chance of lexicalization and the English model a 15% chance. This difference is necessary because the model tends to read lexicalized words faster than pseudo-words. Equating the chance of lexicalization with the size of the database means that the chance a particular word gets lexicalized after a given number of word presentations is similar given otherwise identical conditions. If this change was not used, the English words used for testing (i.e., the experimental stimuli) would have been presented to the network twice as often as the Italian words after the same amount of training.

Creating the models proceeded in a number of steps. First, two monolingual models - called L1 below - were created. This was done by initially training the models on a small set of spelling-sound correspondences to simulate early reading knowledge children have. For the English model, an identical set of correspondences was used as Perry et al. [11]. For the Italian model, 76 grapheme-phoneme correspondences were used. The models were trained on these for 100 cycles. After this, the models were presented with words from the databases 500,000 times, and learning was allowed to occur. Because the results are non-deterministic, this was repeated 10 times and the average results were taken.

Terminology: in what follows, an L1 network is a network that, at the beginning of the simulations, is created and trained with a given -native- language/orthography. For example, the L1-English network is a network that learns to read English first. The same network, once it has learned Italian, will be called L2-Italian. Accordingly, the L2 Italian network is obtained by first *seeding* it with the ortho-phonological weights taken from the L1 English network. At variance with the human data, the L2-networks are not bilingual in a strict sense, as after training in L2 they were exposed to L2 stimuli only, as the network lacks a language recognition and shifting strategy.

Creation of L2-models

After the L1 models were trained, the L2 models were created by taking the connection weights from the spelling-sound mapping network (i.e., the TLA network) of the L1 models at 250.000 cycles and placing them in the corresponding network of the L2 models for all graphemes that were shared across the two languages. Because CDP uses an orthosyllabic template where there are slots for each syllable, this was done in a position-specific manner. In particular, the template used for syllables in the English model consists of 3 possible onset slots, one vowel slot, and 4 possible coda slots (i.e., CCCVCCCC where C represents a consonant grapheme and V a vowel grapheme), and the Italian model has 4 onset slots, one vowel slot, and one coda slot (i.e., CCCCVC). When identical graphemes were found in the first 3 onset positions, the vowel position, and the first coda position, the weights were taken from the grapheme-phoneme connections of the L1 network and copied in the L2 network. This was only done when there was a phoneme that was relatively close in terms of features to the L1 phoneme (a list of these appears in Appendix), including when the phoneme was not or rarely used in the L2 language with the particular grapheme. Weights in slots that were not shared were simply discarded as were graphemes where there was no similar phoneme. This procedure seeds the L2 networks with information from the L1 network and it was done for the first two syllables of both models. Overall, this meant the L2-English model was seeded with 3476 positive and 10893 negative weights, and the L2-Italian model 3522 positive and 10005 negative weights.

Following the seeding of the L2 networks with the L1 weights, the learning procedure was identical to the L1 models, excluding the training on spelling-sound rules and learning rate. Because these are L2 models, we assumed that learning is less plastic for them than the L1 models and we therefore reduced the learning rate which was used in the L2 models from 0.05 to 0.01. The RTs of the models and error rates across training appear in Figure S4.

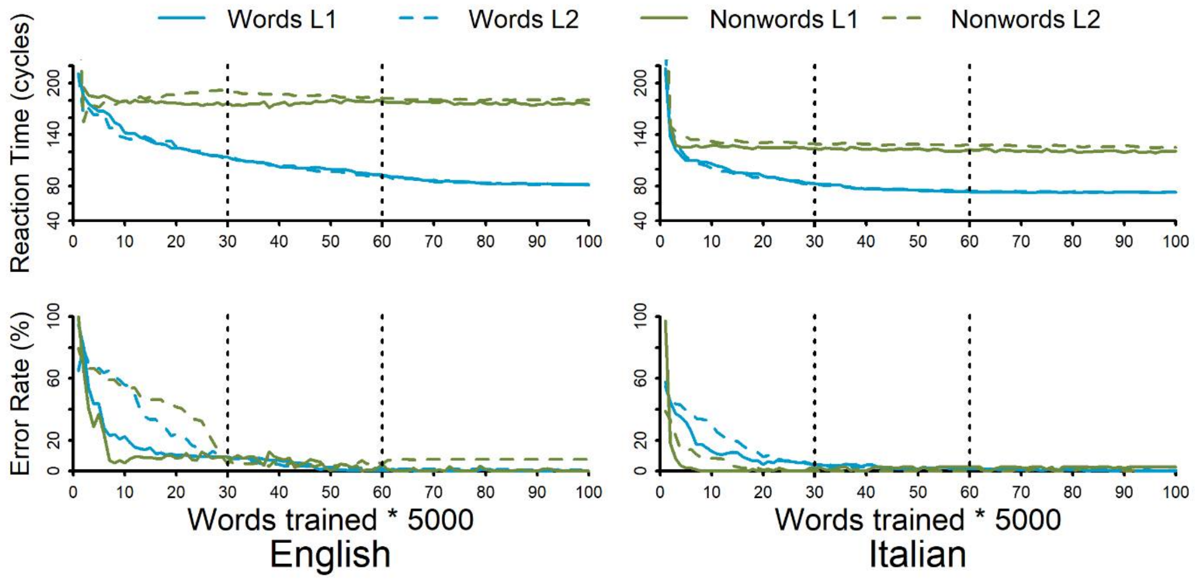

Figure S4. Reaction times and error rates of the models across 500,000 word presentations. Here a L2 model refers to a model that was L1 for English and has then "learned" Italian reading and vice-versa. The vertical dotted lines are the two time-points where the model is examined below (150,000 and 300,000 word presentations).

The results showed that there was an interaction between RTs and lexicality, with the RTs of words dropping throughout training but the RTs of pseudo-words initially dropping but then remaining relatively steady. The RTs of words drop because the TLA networks begin to produce phonology that is closer to the actual words as they are trained more, and also because words become lexicalized which causes them to be read much more quickly. There was also an effect of "bilingualism" and this interacted with lexicality, where the L2 models were slower at reading pseudo-words than the L1 models, but the same was not true of words. This occurred because the effect of less-accurate phonology (e.g., more competitors in the same phoneme position, lower activations etc.) is more on pseudo-words than words. Interestingly, across training, the L1 and L2 pseudo-word RTs converged with the English model, but they did not with the Italian model, even after 500,000 word presentations. This mimics the human data in that Italian and English bilinguals are equally slow when reading English pseudo-words, while Italian bilinguals remain faster when reading Italian pseudo-words. In terms of error rates, there was a main effect of language where both the L1 and L2 English models displayed a higher error rate than the Italian model. There was also an effect of bilingualism where the L2 models displayed a higher error rate than the L1 models, which was greater in English than Italian.

Given the distribution of results over time, we examined the patterns of data the model can produce. We first considered L1 and L2 models trained for 300,000 cycles. At this point in training, all models had a relatively low error rate. The Italian L1 model had an error rate of 1.32% and 2.78%, the Italian L2 model 1.05% and .56%, the English L1 model .80% and 4.35%, and the English L2 model 1.75% and 4.87%, for words and pseudo-words, respectively. The RT results appear in Figure S5.

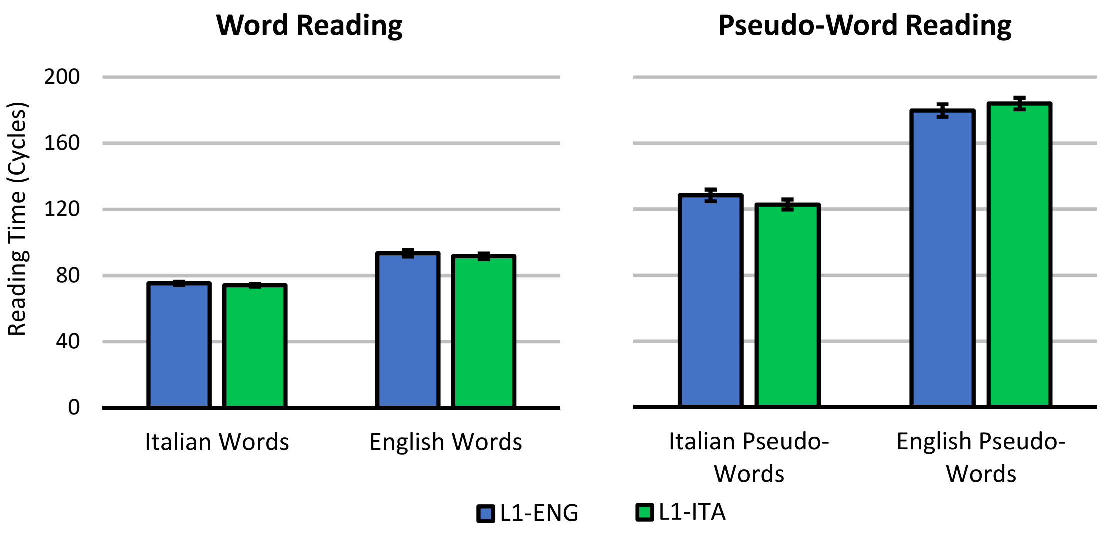

Figure S5. Results of the models after training the L1 and L2 networks on 300,000 word presentations. Error bars are +/- 1SE.

To examine the models' RTs, statistical comparisons using t-tests were performed comparing the L1 and L2 models on the words and pseudowords for each of the two models separately.[1] A difference was found between the Italian words ($t(37) = 2.11$, $p = .042$), although it was not significant after Bonferroni correction. On the other hand, there was a significant difference between the Italian pseudo-words ($t(33) = 3.43$, $p = .0016$), and, in English, no significant difference between the words ($t(39) = 1.01$, $p = .32$) or pseudo-words ($t(36) = 1.41$, $p = .17$).

One aspect of the human data that might offer a potential way for the model to differentiate the L1 and L2 Italian words better is the extent to which learning had occurred in the different groups. Note that learning in the Italian L2 model is faster compared to the English L2 model, thereby suggesting that the latter requires longer training to reach the same level of (bilingual) proficiency. Moreover, in the human data, the L2 English group started learning earlier than the L2 Italian group. We therefore re-examined the simulation data using Italian L2 ("equivalent" to English bilinguals) results from the model at an earlier time, that is after 150,000 word presentations. The results of this simulation appear in Figure 5 in the main manuscript.

The results looked even more like the human data. This was confirmed with t-tests where both the Italian words ($t(37) = 6.71$, $p < .0001$) and pseudo-words ($t(34) = 3.66$, $p = .00085$), showed significant differences.

Seeded versus unseeded weights in L2 models.

To investigate the extent to which the English and Italian TLA networks behave differently, we examined the effect of "bilingualism", or to be exposed and learn a second orthography, on the number of weights in the network. This is important because it can give some insight into how seeding the L2 networks with L1 weights affects performance, as well as overall differences between English and Italian. The negative and positive weights were examined separately because they have quite different distributions and their purpose is different. Positive weights cause phonemes to be activated and increase in size whenever a grapheme and a phoneme co-occur together in training. Negative weights inhibit the phoneme of the grapheme they are connected to; they increase in size when a phoneme is predicted from a given word but is not actually in the word.

In terms of the **number of initial weights that were transferred**, as noted above, both L2 models were very similar, with the English model seeded with 3476 positive and 10893 negative weights, and the Italian model 3522 positive and 10005 negative weights.

That was not the case **later in training**. At 300,000 word presentations (see the values in correspondence of the second vertical dashed lines in both graphs of figure S6), the L2 Italian model had 10023 and 28218 positive and negative weights, and the L2 English model

[1] With the Italian stimuli, two words and five nonwords were removed from the analyses because they were words not in the database and nonwords that were actually low frequency words in the database but unlikely to be known by most Italian speakers. One nonword was removed from the English analyses for the same reason. Please notice that, at variance with the human data, for obvious reasons here only unpaired comparisons were possible.

had 14230 and 44727 positive and negative weights. Thus, the extent of learning not attributable to direct overlap of the L1 and L2 networks was clearly much greater in English than Italian. With the English L2 network, 24.4% of both the positive and negative weights were affected by seeding. With the Italian L2 network, 35.1% and 35.5% of the negative and positive weights were affected. This provides further evidence that the amount of information transferred from the L1 to L2 networks, as function of the total amount of learning that occurred across training, was less when L2 is English than Italian. That is, in the L2 English network, there are many more weights that had their values changed across learning that were not initially affected by seeding from the L1 Italian network.

SUPPLEMENTARY TABLES

Table S4: Frequency data for the proficiency test.
Mean frequency data of the stimuli and associated responses in the proficiency test.

TRIAL TYPE	BLOCK	MEAN STIMULI FREQUENCY (Zipf-SUBTLEX)	MEAN RESPONSE FREQUENCY (Zipf-SUBTLEX)
ITA-to-ENG	BLOCK 1	5.27	5.36
ITA-to-ENG	BLOCK 2	4.32	4.30
ITA-to-ENG	BLOCK 3	3.44	3.34
ENG-to-ITA	BLOCK 1	5.42	5.29
ENG-to-ITA	BLOCK 2	4.86	4.86
ENG-to-ITA	BLOCK 3	4.04	3.92

Table S5: Experimental Stimuli (N-size= Orthographic Neighbourhood size).

TYPE	SERIE	LANGUAGE	ITEM	TYPE SUMMED BIGRAM FREQUENCY	TOKEN SUMMED BIGRAM FREQUENCY (FREQUENCY PER MILLION)	FREQUENCY (SUBTLEX)	FREQUENCY per million (SUBTLEX)	Zipf	N-Size	LETTERS
WORD	BLOCK 1	ITA	TESTA	91982	174932.89	41400	445.14	5.65	27	5
WORD	BLOCK 1	ITA	GOCCIA	60838	95281.90	988	10.62	4.03	7	6
WORD	BLOCK 1	ITA	UOVO	11345	44414.66	1461	15.71	4.20	19	4
WORD	BLOCK 1	ITA	NEVE	30947	64552.73	2802	30.13	4.48	20	4
WORD	BLOCK 1	ITA	COLLO	60543	136598.85	5620	60.43	4.78	25	5
WORD	BLOCK 1	ITA	SPADA	28919	56688.09	4367	46.95	4.67	7	5
WORD	BLOCK 1	ITA	TRENO	88461	214857.36	6543	70.35	4.85	16	5
WORD	BLOCK 1	ITA	FILO	28795	59305.44	2726	29.31	4.47	39	4
WORD	BLOCK 1	ITA	BARBA	49835	64843.92	1832	19.70	4.29	11	5
WORD	BLOCK 1	ITA	CANE	70563	101829.55	13494	145.09	5.16	38	4
WORD	BLOCK 1	ITA	ARCO	61860	114278.97	963	10.35	4.01	19	4
WORD	BLOCK 1	ITA	STRADA	75689	144988.55	26412	283.99	5.45	10	6
WORD	BLOCK 1	ITA	FERRO	71003	113236.88	2238	24.06	4.38	16	5
WORD	BLOCK 1	ITA	VENTO	78188	174583.61	4427	47.60	4.68	25	5
WORD	BLOCK 1	ITA	MURO	27191	38549.63	5390	57.95	4.76	41	4
WORD	BLOCK 1	ITA	FIORE	74131	157892.08	1764	18.97	4.28	8	5
WORD	BLOCK 1	ITA	RUOTA	39355	75782.78	1591	17.11	4.23	10	5
WORD	BLOCK 1	ITA	PALLA	57582	120235.23	6031	64.85	4.81	33	5
WORD	BLOCK 1	ITA	CIBO	21784	32100.04	11354	122.08	5.09	33	4
WORD	BLOCK 1	ITA	MOTO	42530	101181.86	3318	35.68	4.55	49	4

WORD	BLOCK 2	ITA	LETTO	67037	146944.54	33203	357.00	5.55	27	5
WORD	BLOCK 2	ITA	PACCO	51359	100875.52	1754	18.86	4.27	19	5
WORD	BLOCK 2	ITA	TERRA	109215	170907.77	26304	282.82	5.45	29	5
WORD	BLOCK 2	ITA	CODA	31513	80133.69	2234	24.02	4.38	42	4
WORD	BLOCK 2	ITA	PIEDE	36141	77565.58	4770	51.29	4.71	15	5
WORD	BLOCK 2	ITA	FRUTTA	53649	101786.28	1721	18.50	4.27	11	6
WORD	BLOCK 2	ITA	SOLE	39744	89701.75	9987	107.38	5.03	35	4
WORD	BLOCK 2	ITA	BANCO	69375	120159.09	1681	18.07	4.26	15	5
WORD	BLOCK 2	ITA	LAGO	29227	55037.81	3003	32.29	4.51	32	4
WORD	BLOCK 2	ITA	PIANTA	114747	190425.80	1213	13.04	4.11	9	6
WORD	BLOCK 2	ITA	TOPO	33219	92595.87	1789	19.24	4.28	27	4
WORD	BLOCK 2	ITA	SEDIA	55741	135509.64	4290	46.13	4.66	8	5
WORD	BLOCK 2	ITA	BOCCA	37973	47738.43	11851	127.42	5.10	14	5
WORD	BLOCK 2	ITA	LUNA	24271	64789.13	6539	70.31	4.85	30	4
WORD	BLOCK 2	ITA	CARTA	88629	137270.88	8741	93.98	4.97	24	5
WORD	BLOCK 2	ITA	GRANO	85391	171067.65	925	9.95	4.00	24	5
WORD	BLOCK 2	ITA	BORSA	38115	79745.01	7169	77.08	4.89	14	5
WORD	BLOCK 2	ITA	PONTE	87609	175516.44	5049	54.29	4.73	16	5
WORD	BLOCK 2	ITA	DISCO	63621	137633.87	2133	22.93	4.36	15	5
WORD	BLOCK 2	ITA	NASO	38381	77063.22	5593	60.14	4.78	32	4
PSEUDO	BLOCK 1	ITA	FIOMO	43721	87234.54				7	5
PSEUDO	BLOCK 1	ITA	SPASO	38707	77371.32				8	5
PSEUDO	BLOCK 1	ITA	RALA	65935	120548.73				34	4
PSEUDO	BLOCK 1	ITA	TENZA	64690	107375.77				15	5
PSEUDO	BLOCK 1	ITA	RIDIA	74850	132978.83				11	5
PSEUDO	BLOCK 1	ITA	SCARO	84228	119953.50				15	5
PSEUDO	BLOCK 1	ITA	BRAZZA	48569	71477.83				1	6
PSEUDO	BLOCK 1	ITA	TORLA	67282	155839.13				16	5
PSEUDO	BLOCK 1	ITA	MARO	67316	109426.82				46	4

PSEUDO	BLOCK 1	ITA	NACIA	66858	108541.35	9	5
PSEUDO	BLOCK 1	ITA	SELE	42610	90748.20	29	4
PSEUDO	BLOCK 1	ITA	BAMO	35809	59808.57	31	4
PSEUDO	BLOCK 1	ITA	ERPO	52350	94555.45	14	4
PSEUDO	BLOCK 1	ITA	TESCA	76316	120811.23	5	5
PSEUDO	BLOCK 1	ITA	MONSO	56809	138999.09	9	5
PSEUDO	BLOCK 1	ITA	PRAMO	68451	119508.66	12	5
PSEUDO	BLOCK 1	ITA	SAGO	20200	42885.62	34	4
PSEUDO	BLOCK 1	ITA	PATRA	83297	126969.44	6	5
PSEUDO	BLOCK 1	ITA	STOMO	63397	151964.91	11	5
PSEUDO	BLOCK 1	ITA	TERBA	81257	126239.26	15	5
PSEUDO	BLOCK 2	ITA	BANSA	56228	91053.03	10	5
PSEUDO	BLOCK 2	ITA	LADE	36648	82794.56	24	4
PSEUDO	BLOCK 2	ITA	NEBRO	40639	67538.03	2	5
PSEUDO	BLOCK 2	ITA	PAVE	27153	58915.16	22	4
PSEUDO	BLOCK 2	ITA	LUGNO	24915	84967.99	3	5
PSEUDO	BLOCK 2	ITA	FRADA	45093	79061.37	5	5
PSEUDO	BLOCK 2	ITA	NUSO	15717	45331.29	14	4
PSEUDO	BLOCK 2	ITA	STRACO	94531	183067.67	2	6
PSEUDO	BLOCK 2	ITA	MOBA	21927	36292.62	17	4
PSEUDO	BLOCK 2	ITA	CIVE	30538	58969.87	16	4
PSEUDO	BLOCK 2	ITA	FIBA	15059	18183.63	16	4
PSEUDO	BLOCK 2	ITA	OLSA	25478	54955.33	13	4
PSEUDO	BLOCK 2	ITA	FOLO	31301	58738.92	28	4
PSEUDO	BLOCK 2	ITA	ARBA	43736	57803.80	16	4
PSEUDO	BLOCK 2	ITA	VADIO	44061	110388.17	6	5
PSEUDO	BLOCK 2	ITA	FIME	29015	49126.16	22	4
PSEUDO	BLOCK 2	ITA	OMBA	17524	27130.49	7	4
PSEUDO	BLOCK 2	ITA	RONE	64080	131793.08	42	4

PSEUDO	BLOCK 2	ITA	BORTA	58695	107931.58				16	5
PSEUDO	BLOCK 2	ITA	VIBA	17423	24947.96				20	4
WORD	RANDOM 1	ITA	OSSO	32429	86548.93	1939	20.85	4.32	16	4
WORD	RANDOM 1	ITA	DENTE	90309	164456.78	1036	11.14	4.05	18	5
WORD	RANDOM 1	ITA	ORTO	50539	121806.11	222	2.39	3.38	27	4
WORD	RANDOM 1	ITA	MARE	81401	149895.97	6919	74.39	4.87	46	4
WORD	RANDOM 1	ITA	ERBA	51446	79981.59	3633	39.06	4.59	21	4
WORD	RANDOM 1	ITA	CALZA	41873	63581.37	233	2.51	3.40	16	5
WORD	RANDOM 1	ITA	MONTE	94036	180371.68	1599	17.19	4.23	20	5
WORD	RANDOM 1	ITA	PRATO	90990	172504.83	944	10.15	4.01	10	5
WORD	RANDOM 1	ITA	SUCCO	38908	88301.99	1789	19.24	4.28	8	5
WORD	RANDOM 1	ITA	PINO	54853	125432.76	447	4.81	3.68	33	4
WORD	RANDOM 1	ITA	VISO	33520	66012.07	4004	43.05	4.63	25	4
WORD	RANDOM 1	ITA	PORTA	62837	127370.80	38372	412.58	5.61	28	5
WORD	RANDOM 1	ITA	FRUTTO	48875	117659.74	964	10.37	4.01	13	6
WORD	RANDOM 1	ITA	CASA	41383	69608.73	129959	1397.34	6.14	46	4
WORD	RANDOM 1	ITA	ACQUA	18044	60016.14	22228	239.00	5.38	9	5
WORD	RANDOM 2	ITA	CORPO	50321	120017.32	21105	226.92	5.35	17	5
WORD	RANDOM 2	ITA	TETTO	80437	162871.40	4144	44.56	4.65	34	5
WORD	RANDOM 2	ITA	ROSA	38241	87952.19	3701	39.79	4.60	47	4

WORD	RANDOM 2	ITA	LATTE	94235	161347.49	4456	47.91	4.68	27	5
WORD	RANDOM 2	ITA	SEGA	24983	47747.11	1187	12.76	4.10	39	4
WORD	RANDOM 2	ITA	STOFFA	50910	132314.27	540	5.81	3.76	2	6
WORD	RANDOM 2	ITA	FUOCO	30943	79119.87	16570	178.16	5.25	7	5
WORD	RANDOM 2	ITA	CORDA	53301	126497.49	2454	26.39	4.42	18	5
WORD	RANDOM 2	ITA	RIVA	45779	58389.74	898	9.66	3.98	32	4
WORD	RANDOM 2	ITA	PIETRA	74660	121515.74	3020	32.47	4.51	4	6
WORD	RANDOM 2	ITA	GATTO	73908	145453.65	4110	44.19	4.64	21	5
WORD	RANDOM 2	ITA	CARNE	74385	108743.34	6665	71.66	4.85	21	5
WORD	RANDOM 2	ITA	MANO	65604	149207.28	31080	334.18	5.52	49	4
WORD	RANDOM 2	ITA	CROCE	36660	59824.27	1849	19.88	4.30	4	5
WORD	RANDOM 2	ITA	COLLA	66435	150881.46	616	6.62	3.82	27	5
WORD	BLOCK 1	ENG	NEEDLE	37632	88934.85	1709	8.49	3.93	4	6
WORD	BLOCK 1	ENG	CHERRY	41733	184793.49	3398	16.88	4.23	3	6
WORD	BLOCK 1	ENG	BATTLE	32049	85645.66	17431	86.58	4.94	12	6
WORD	BLOCK 1	ENG	PAPER	34035	83405.24	17429	86.57	4.94	18	5
WORD	BLOCK 1	ENG	WINDOW	36760	156664.06	13791	68.50	4.84	2	6
WORD	BLOCK 1	ENG	DOCTOR	23730	95725.93	21190	105.25	5.02	2	6
WORD	BLOCK 1	ENG	APPLE	18842	41600.33	7734	38.41	4.58	3	5
WORD	BLOCK 1	ENG	SILVER	38989	117600.94	20779	103.21	5.01	4	6
WORD	BLOCK 1	ENG	FOREST	51430	166666.58	9388	46.63	4.67	1	6
WORD	BLOCK 1	ENG	PUPPY	4751	13028.24	1441	7.16	3.85	9	5
WORD	BLOCK 1	ENG	ROCKET	23801	58737.74	4252	21.12	4.32	9	6

WORD	BLOCK 1	ENG	JELLY	20761	60916.87	3600	17.88	4.25	18	5
WORD	BLOCK 1	ENG	TRUMPET	23293	49159.65	1321	6.56	3.82	3	6
WORD	BLOCK 1	ENG	LADDER	43955	99177.19	2934	14.57	4.16	12	6
WORD	BLOCK 1	ENG	CABIN	35235	104418.73	1593	7.91	3.90	10	5
WORD	BLOCK 1	ENG	MOTOR	22987	97610.73	3551	17.64	4.25	7	5
WORD	BLOCK 1	ENG	PONY	18021	53399.71	1824	9.06	3.96	25	4
WORD	BLOCK 1	ENG	STABLE	37344	83911.39	4062	20.18	4.30	4	6
WORD	BLOCK 1	ENG	CHIMNEY	26422	84396.04	1609	7.99	3.90	0	6
WORD	BLOCK 1	ENG	RADISH	35048	82744.28	332	1.65	3.22	3	6
WORD	BLOCK 2	ENG	ANKLE	29258	88269.94	1767	8.78	3.94	5	5
WORD	BLOCK 2	ENG	MIRROR	27847	72985.59	5672	28.17	4.45	0	6
WORD	BLOCK 2	ENG	BUNNY	10790	26562.61	1566	7.78	3.89	20	5
WORD	BLOCK 2	ENG	KENNEL	34706	89240.04	445	2.21	3.35	9	6
WORD	BLOCK 2	ENG	FABRIC	25144	49025.75	1898	9.43	3.97	1	6
WORD	BLOCK 2	ENG	CANDY	27882	112588.53	1360	6.75	3.83	22	5
WORD	BLOCK 2	ENG	PLANET	39963	117508.03	9209	45.74	4.66	3	6
WORD	BLOCK 2	ENG	LOCKER	38214	96605.37	803	3.99	3.60	14	6
WORD	BLOCK 2	ENG	BUBBLE	20259	41800.36	2924	14.52	4.16	11	6
WORD	BLOCK 2	ENG	SOFA	5848	41798.24	3341	16.59	4.22	21	4
WORD	BLOCK 2	ENG	ARMY	14570	39025.01	16200	80.46	4.91	14	4
WORD	BLOCK 2	ENG	TABLE	25831	51730.58	25504	126.67	5.10	10	5
WORD	BLOCK 2	ENG	KETTLE	26383	63226.69	2105	10.46	4.02	4	6
WORD	BLOCK 2	ENG	TUNNEL	26469	59201.46	3455	17.16	4.23	3	6
WORD	BLOCK 2	ENG	POCKET	19258	47396.81	8690	43.16	4.63	8	6
WORD	BLOCK 2	ENG	BERRY	31795	93142.76	1262	6.27	3.80	19	5
WORD	BLOCK 2	ENG	NAPKIN	33994	93039.53	411	2.04	3.31	1	6
WORD	BLOCK 2	ENG	PENCIL	28848	71742.04	1921	9.54	3.98	0	6
WORD	BLOCK 2	ENG	COLLAR	40716	102785.09	1912	9.50	3.98	7	6
WORD	BLOCK 2	ENG	VILLAGE	31904	80044.17	16775	83.32	4.92	2	7

PSEUDO	BLOCK 1	ENG	RIFFEN	27473	68069.52	1	6
PSEUDO	BLOCK 1	ENG	SOTA	12783	46010.07	30	4
PSEUDO	BLOCK 1	ENG	BAMMEL	23875	62927.24	3	6
PSEUDO	BLOCK 1	ENG	CODDAR	25702	66383.96	0	6
PSEUDO	BLOCK 1	ENG	TRUDLET	27664	56081.09	0	6
PSEUDO	BLOCK 1	ENG	NASLIN	45531	128892.77	2	6
PSEUDO	BLOCK 1	ENG	STAFEL	28858	71820.69	0	6
PSEUDO	BLOCK 1	ENG	MENAL	33716	94225.36	8	5
PSEUDO	BLOCK 1	ENG	BURRON	32150	90119.31	7	6
PSEUDO	BLOCK 1	ENG	PABER	33940	94013.49	14	5
PSEUDO	BLOCK 1	ENG	LEGON	29547	83003.00	9	5
PSEUDO	BLOCK 1	ENG	BEPPY	8337	28739.41	7	5
PSEUDO	BLOCK 1	ENG	KEFFLE	20517	47203.09	0	6
PSEUDO	BLOCK 1	ENG	SIMNER	39396	100913.84	6	6
PSEUDO	BLOCK 1	ENG	ROBBET	20206	59403.58	5	6
PSEUDO	BLOCK 1	ENG	MOPOR	19332	57652.08	2	5
PSEUDO	BLOCK 1	ENG	KEPPLE	22014	53578.47	0	6
PSEUDO	BLOCK 1	ENG	PITTOW	21967	107564.52	0	6
PSEUDO	BLOCK 1	ENG	ARBY	14061	35049.69	14	4
PSEUDO	BLOCK 1	ENG	BUFFLE	18280	40334.40	4	6
PSEUDO	BLOCK 2	ENG	BONNEL	36045	93100.34	5	6
PSEUDO	BLOCK 2	ENG	RANISH	46320	127701.98	8	6
PSEUDO	BLOCK 2	ENG	SAMIN	35898	101701.45	18	5
PSEUDO	BLOCK 2	ENG	WIMPOW	13337	49642.22	0	6
PSEUDO	BLOCK 2	ENG	FASRIC	25014	63043.61	2	6
PSEUDO	BLOCK 2	ENG	BOFFY	6399	34568.64	3	5
PSEUDO	BLOCK 2	ENG	HAPPER	38883	127544.48	17	6
PSEUDO	BLOCK 2	ENG	TRAFLOR	33176	73289.46	0	6
PSEUDO	BLOCK 2	ENG	MONLEY	32862	87167.20	9	6

PSEUDO	BLOCK 2	ENG	AFTON	21869	84556.52				9	5
PSEUDO	BLOCK 2	ENG	RABOR	27051	61453.65				6	5
PSEUDO	BLOCK 2	ENG	CAFLY	13171	33503.90				4	5
PSEUDO	BLOCK 2	ENG	BUPPY	5858	19096.06				8	5
PSEUDO	BLOCK 2	ENG	CODDER	40725	101801.59				12	6
PSEUDO	BLOCK 2	ENG	RANNITT	43636	131163.91				0	6
PSEUDO	BLOCK 2	ENG	TIPER	41181	96631.40				14	5
PSEUDO	BLOCK 2	ENG	PEMMY	10165	27589.14				6	5
PSEUDO	BLOCK 2	ENG	BUCKER	34208	92291.58				18	6
PSEUDO	BLOCK 2	ENG	CALLOT	33042	104132.48				7	6
PSEUDO	BLOCK 2	ENG	ADDOW	10852	41773.14				3	5
WORD	RANDOM 1	ENG	TIGER	41527	104299.67	4601	22.85	4.36	13	5
WORD	RANDOM 1	ENG	CANNON	40486	136669.57	2404	11.94	4.08	5	6
WORD	RANDOM 1	ENG	BUTTON	28877	113600.07	5813	28.87	4.46	13	6
WORD	RANDOM 1	ENG	BUCKET	17181	49779.73	3295	16.37	4.21	2	6
WORD	RANDOM 1	ENG	TRACTOR	38853	111883.16	1404	6.97	3.84	3	6
WORD	RANDOM 1	ENG	SLIPPER	42144	97700.16	314	1.56	3.19	7	6
WORD	RANDOM 1	ENG	ORGAN	29138	95681.50	1948	9.68	3.99	7	5
WORD	RANDOM 1	ENG	MUSTARD	39313	102218.82	2345	11.65	4.07	2	6
WORD	RANDOM 1	ENG	RIBBON	29739	70206.91	765	3.80	3.58	1	6
WORD	RANDOM 1	ENG	MARKET	30325	78592.92	33772	167.74	5.22	7	6
WORD	RANDOM 1	ENG	TICKET	31974	74485.43	6464	32.11	4.51	6	6
WORD	RANDOM 1	ENG	PEPPER	36643	91996.07	4855	24.11	4.38	5	6

WORD	RANDOM 1	ENG	SADDLE	22143	44951.97	1231	6.11	3.79	6	6
WORD	RANDOM 1	ENG	RAZOR	21264	46233.25	978	4.86	3.69	3	5
WORD	RANDOM 1	ENG	BASKET	20527	62952.70	2931	14.56	4.16	7	6
WORD	RANDOM 2	ENG	VALLEY	34878	92614.35	6003	29.82	4.47	11	6
WORD	RANDOM 2	ENG	APRON	27675	73507.73	608	3.02	3.48	8	5
WORD	RANDOM 2	ENG	CARROT	32515	88078.13	2410	11.97	4.08	6	6
WORD	RANDOM 2	ENG	BOTTLE	23842	60037.69	8898	44.19	4.65	12	6
WORD	RANDOM 2	ENG	MONKEY	25309	79803.68	4848	24.08	4.38	4	6
WORD	RANDOM 2	ENG	ELBOW	14275	41841.25	1440	7.15	3.85	2	5
WORD	RANDOM 2	ENG	BARREL	40348	117659.44	2305	11.45	4.06	10	6
WORD	RANDOM 2	ENG	RATTLE	38643	91530.38	808	4.01	3.60	6	6
WORD	RANDOM 2	ENG	COPPER	39081	102841.43	3620	17.98	4.25	14	6
WORD	RANDOM 2	ENG	COFFEE	15897	68306.82	9060	45.00	4.65	3	6
WORD	RANDOM 2	ENG	TEMPLE	32218	72233.23	2160	10.73	4.03	2	6
WORD	RANDOM 2	ENG	PILLOW	24354	76948.89	1077	5.35	3.73	4	6
WORD	RANDOM 2	ENG	HELMET	26518	158346.26	2398	11.91	4.08	4	6
WORD	RANDOM 2	ENG	PEDAL	29711	71207.05	868	4.31	3.63	4	5
WORD	RANDOM 2	ENG	CHICKEN	38941	112077.59	13434	66.72	4.82	4	6

Table S6: Normality tests for the control tasks.

	VOCAL REACTION TIME	ARTICULATIO N SPEED	FLUENCY (ITA)	FLUENCY (ENG)	STROOP EFFECT ITALIAN	STROOP EFFECT ENGLISH
N	40	40	40	40	40	40
Shapiro-Wilk p	0.317	0.135	0.298	0.890	0.048	0.564

Table S7: Cross-linguistic comparison of psycholinguistic data for experimental stimuli.

Independent Samples Mann-Whitney tests

**Words**		**Statistic**	**p**
N-Size	Mann-Whitney U	565.500	< .001
TOKEN SUMMED BIGRAM FREQUENCY (FREQUENCY PER MILLION)	Mann-WhitneyU	1586.000	< .001
TYPE SUMMED BIGRAM FREQUENCY	Mann-Whitney U	762.000	< .001
Zipf	Mann-Whitney U	1376.000	< .001

**Pseudo-Words**		**Statistic**	**p**
N-Size	Mann-Whitney U	345.500	< .001
TOKEN SUMMED BIGRAM FREQUENCY (FREQUENCY PER MILLION)	Mann-Whitney U	612.000	0.071
TYPE SUMMED BIGRAM FREQUENCY	Mann-Whitney U	332.000	< .001

Table S8: % accurate trials in all experimental conditions.

		**Words (Blocked order)**		**Words (Mixed order)**		**Pseudo-words (Blocked order)**	
**Language**		**ITA**	**ENG**	**ITA**	**ENG**	**ITA**	**ENG**
**Group**	**L1-ENG**	**99.639**	**99.525**	**99.209**	**99.846**	**99.410**	**99.193**
	**L1-ITA**	**97.974**	**99.854**	**98.839**	**98.627**	**98.718**	**99.116**

Section

Grapheme-phoneme correspondences used to seed the L2 networks. Italian phonemes are those used in [12] and English phonemes are those used in the CELEX database [13].

**Italian L1 to English L2**			**English L1 to Italian L2**		
Example	*Italian*	*English*	*Example*	*English*	*Italian*
i**n**vece	F	X	f**or**t	$	X
de**gl**i	L	X	fath**er**	@	X
pr**i**ma	i	I	bay	1	X
mor**e**	e	E	bite	2	X
nonch**e**	E	E	herd	3	X
**a**ltro	a	{	boil	4	X
m**o**ndo	o	Q	boat	5	X
m**o**re	O	Q	tout	6	X
n**u**mero	u	V	pier	7	X
p**i**azza	j	j	pear	8	X
q**u**ando	w	w	pure	9	X
**p**rima	p	p	m**ee**t	i	X
**t**empo	t	t	**h**it	h	X
**c**omune	k	k	**th**eir	D	T
itter**b**ii	b	b	H**e**n	E	e
**d**ovessi	d	d	H**i**t	I	i
**g**rande	g	g	**ch**ips	J	S
ste**f**ano	f	f	Si**ng**	N	N
**s**tate	s	s	h**o**t	Q	o
**c**erca	S	S	**Sh**ip	S	S
**V**ive	v	v	**th**ink	T	T
mu**s**ica	z	z	f**oo**t	U	u
**m**ondo	m	m	b**u**t	V	u
**n**ome	n	n	Vi**s**ual	Z	_
**g**iorni	Z	_	**b**ug	b	b
sen**z**a	T	D	**f**ug	d	d
**z**ona	_	z	**f**it	f	f
biso**gn**o	J	X	**g**it	g	g
**g**enere	Z	_	**y**ak	j	j
a**n**cora	N	N	**k**ick	k	k
c**l**audio	l	l	**l**ick	l	l
Lib**r**o	r	r	**m**uck	m	m

Note: X = No translation

Supplementary references

1. Perry, C.; Ziegler, J. C.; Zorzi, M., CDP++. Italian: Modelling sublexical and supralexical inconsistency in a shallow orthography. *PloS one* **2014,** *9* (4), e94291.
2. Perry, C.; Ziegler, J. C.; Zorzi, M., A computational and empirical investigation of graphemes in reading. *Cogn Sci* **2013,** *37* (5), 800-28.
3. van Heuven, W. J.; Mandera, P.; Keuleers, E.; Brysbaert, M., SUBTLEX-UK: a new and improved word frequency database for British English. *Q J Exp Psychol (Hove)* **2014,** *67* (6), 1176-90.
4. Rastle, K.; Davis, M. H., On the complexities of measuring naming. *Journal of Experimental Psychology: Human Perception and Performance* **2002,** *28* (2), 307.
5. Coltheart, M.; Davelaar, E.; Jonasson, J. T.; Besner, D., Access to the internal lexicon. S. Dornick (ed.) Attention and performance, volume VI, 535–556. Hillsdale, New Jersey: Erlbaum: 1977.
6. Schlösser, R.; Hutchinson, M.; Joseffer, S.; Rusinek, H.; Saarimaki, A.; Stevenson, J.; Dewey, S. L.; Brodie, J. D., Functional magnetic resonance imaging of human brain activity in a verbal fluency task. *J Neurol Neurosurg Psychiatry* **1998,** *64* (4), 492-8.
7. Godefroy, O., Frontal syndrome and disorders of executive functions. *J Neurol* **2003,** *250* (1), 1-6.
8. MacLeod, C. M., Half a century of research on the Stroop effect: an integrative review. *Psychol Bull* **1991,** *109* (2), 163-203.
9. Bench, C. J.; Frith, C. D.; Grasby, P. M.; Friston, K. J.; Paulesu, E.; Frackowiak, R. S.; Dolan, R. J., Investigations of the functional anatomy of attention using the Stroop test. *Neuropsychologia* **1993,** *31* (9), 907-22.
10. Perry, C.; Zorzi, M.; Ziegler, J. C., Understanding dyslexia through personalized large-scale computational models. *Psychological science* **2019,** *30* (3), 386-395.
11. Perry, C., Reading Orthographically Strange Nonwords: Modelling Backup Strategies in Reading. *Scientific Studies of Reading* **2018,** *22* (3), 264-272.
12. Adsett, C. R.; Marchand, Y., Syllabic complexity: A computational evaluation of nine European languages. *Journal of Quantitative Linguistics* **2010,** *17* (4), 269-290.
13. Baayen, R. H.; Piepenbrock, R.; Gulikers, L., The CELEX lexical database (cd-rom). **1996**.
